# Evolution of Short‐Range Order of Amorphous GeTe Upon Structural Relaxation Obtained by TEM Diffractometry and RMC Methods

**DOI:** 10.1002/advs.202304323

**Published:** 2023-10-31

**Authors:** Christian Stenz, Julian Pries, T. Wesley Surta, Michael W. Gaultois, Matthias Wuttig

**Affiliations:** ^1^ Institute of Physics IA RWTH Aachen University 52074 Aachen Germany; ^2^ University of Liverpool Liverpool L69 3BX UK; ^3^ Leverhulme Research Centre for Functional Materials Design University of Liverpool, Materials Innovation Factory Liverpool L69 7ZD UK; ^4^ Peter Grünberg Institute (PGI 10) Forschungszentrum Jülich GmbH 52425 Jülich Germany

**Keywords:** metavalent bonding, pair distribution function, phase change materials, resistance drift, reverse Monte‐Carlo simulation, structural relaxation, structure function, transmission electron microscope diffraction

## Abstract

Glasses frequently reveal structural relaxation that leads to changes in their physical properties including enthalpy, specific volume, and resistivity. Analyzing the short‐range order (SRO) obtained from electron diffraction by transmission electron microscopy (TEM) in combination with Reverse‐Monte‐Carlo (RMC) simulations is shown to provide information on the atomic arrangement. The technique elaborated here features several benefits including reliability, accessibility, and allows for obtaining detailed structural data quickly. This is demonstrated with a detailed view of the structural changes in the as‐deposited amorphous phase change material (PCM) GeTe. The data show a significant increase in the average bond angle upon thermal treatment. At the same time the fraction of tetrahedrally coordinated Ge atoms decreases due to an increase in octahedrally distorted and pyramidal motifs. This finding provides further evidence for the atomic processes that govern structural relaxation in amorphous GeTe and other PCMs. A thorough literature review finally unveils possible origins of the large discrepancies reported on the structure of amorphous GeTe.

## Graphical abstract

Structure function ϕ(q) and Pair Distribution Function G(r) of the technologically relevant phase‐change‐material GeTe are obtained by TEM diffraction. Reverse‐Monte‐Carlo simulations based on this structural data reveal atomic rearrangement processes, shedding light on structural relaxation that leads to resistance drift in GeTe. The here elaborated method is capable of reliably reproducing structural models obtained from large scale synchrotron‐facilities as shown for several materials like amorphous silica.

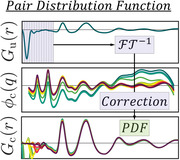

## 1 Introduction

### 1.1 The Quest for Fast but Accurate Structural Studies

Studies of the atomic arrangement based on pair distribution function (PDF) analysis have proven to be a powerful tool to understand the properties of functional materials.^[^ ^1^ ^]^ Some of the key characteristics of incipient metals,^[^ ^2^ ^]^ which often provide a superior performance in thermoelectric applications or as a phase‐change material (PMC), are largely governed by the atomic short‐range order (SRO).^[^ ^3^, ^4^, ^5^ ^]^ The most common technique for accessing the local atomic structure of such materials experimentally is by X‐ray and neutron total scattering. Clearly, the advantage of a synchrotron or neutron beam line experiment is the high resolution in reciprocal space and the high scattering angles that can be achieved compared to conventional transmission electron microscopes. Furthermore, there is already well‐elaborated analysis software available from which essential functions like the structure function and the PDF can be computed. On the other hand, beam time is rare due to the high demand in high precision measurements from the community.

Recent work has shown that selected area electron diffraction (SAED) patterns acquired at transmission electron microscopes (TEMs) can also be employed for PDF studies.^[^ ^6^ ^]^ That brings numerous advantages with it: Besides the much wider availability of TEMs in comparison to any beam line, smaller probing areas within the sample can be accessed, allowing for the examination of structural fluctuations within a sample, not only from resulting electron PDFs (ePDFs) but also in combination with real space TEM images of the area probed.

Nevertheless, there are also limitations to ePDF: To obtain a physically correct structure function *S*(*q*) from the diffraction data (with $q=|\vec{q}|$ being the length of the reciprocal scattering vector), multiple scattering and inelastic scattering of the probing electrons have to be minimized. Multiple and inelastic scattering lead to a *q*‐dependent background in the diffraction pattern that cannot be removed properly, e.g., by fitting element based atomic form factors. Inelastic scattering can be minimized using an energy filter in TEM. Inelastic scattering and multiple scattering are additionally reduced by utilizing samples that are much thinner than the electron mean free path in the material examined. Yet, not all TEM setups provide an energy filter as a standard feature and there are materials that are affected by reducing the sample size. Hence, these requirements often cannot be perfectly satisfied. Additionally, it is unclear how the instrumental setup may introduce a deviation from the expected background function.^[^ ^6^ ^]^ These disruptive factors are manifested in a systematic deviation of the background fit (which relies on element based atomic form factors). In other structural studies, these undesired contributions have been corrected for by fitting additional empirical models like a polynomial to the structure function *S*(*q*) or a Laurent‐type series to the diffraction profile, making a quantitative analysis of the PDF questionable.^[^ ^7^, ^8^ ^]^

To enhance the reliability of a quantitative analysis, in the present work a self‐consistent technique is introduced to correct for a low frequency distortion in structure function *S*(*q*), which is combined with Reverse‐Monte‐Carlo (RMC) simulations. This distortion is mainly caused by inelastic scattering. The validity and limitations of the technique employed are verified by RMC simulations on glassy SiO_2_, which serves as a standard. Subsequently, we study the SRO of a characteristic PCM, namely GeTe.

### 1.2 PCM Applications and Resistance Drift

PCMs are employed in fast and non‐volatile optical and electrical data storage devices. PCMs show *inter alia* high optical and electrical property contrasts between their amorphous and crystalline phase, which are both accessible through high‐speed phase transitions. In a binary PCM‐device a bit of information can be stored by crystallizing (writing a logical “1”) or amorphizing (writing a logical “0” into) a PCM‐cell, *e. g*., by laser crystallization and laser melt‐quenching. These PCM‐cells can afterwards be read‐out electrically or optically as a “0” or “1”, due to the stark property contrast. Non‐volatility of a PCM‐device is also ensured since the meta‐stable amorphous phase (as well as the crystalline phase) is retained at room temperature for at least several decades.^[^ ^9^ ^]^

However, thermal treatment induces structural relaxation of (glassy) amorphous GeTe (a‐GeTe) leading to a lowering in enthalpy^[^ ^10^ ^]^ or an increase in resistivity^[^ ^11^, ^12^ ^]^ (so‐called resistance drift). These structural changes are investigated qualitatively by the order parameters *S*(*q*_2_)/*S*(*q*_1_) and *r*_2_/*r*_1_, relating the first two peaks' heights *S*(*q*_*i*_) in the structure function and the first two peak positions *r*_*i*_ in the PDF, respectively.^[^ ^13^, ^14^ ^]^ Usually, these order parameters are utilized to determine the predominant structural motifs in disordered materials, *i. e*. if they are octahedrally coordinated (*S*(*q*_2_)/*S*(*q*_1_) < 1 and *r*_2_/*r*_1_ ≈ 1.41) or tetrahedrally coordinated (*S*(*q*_2_)/*S*(*q*_1_) > 1 and *r*_2_/*r*_1_ ≈ 1.63). Subsequent to the qualitative analysis of the structural changes in a‐GeTe, RMC simulations for a (semi‐)quantitative insight have been employed. Applying RMC methods allows for the generation of a 3D atomic model taking into account the different types of atomic species. Such a model can then be analyzed in terms of structural motifs, bond angle distributions, or coordination numbers. The combined approach of ePDF and RMC allows for a detailed and reliable insight into the structural relaxation mechanisms. The contrast in resistance between the amorphous and crystalline phase in GeTe and other PCMs is so large that the resistance drift of the amorphous phase is not disturbing a correct read‐out in binary memory devices. The wide resistance range between amorphous and crystalline GeTe is highly attractive for multi‐level systems,^[^ ^15^ ^]^ realized by tuning the crystalline‐to‐amorphous volume fraction in a PCM‐device. This has been perceived as a chance to mimic synaptic weights and contribute to the advancement of neuromorphic computing.^[^ ^16^, ^17^, ^18^ ^]^ However, in PCM‐based multi‐level devices resistance drift will lead to a corrupted read‐out after a certain time interval has passed.^[^ ^19^ ^]^ Therefore, one of the challenges^[^ ^20^ ^]^ is the fundamental understanding and controlling of the resistance drift in amorphous PCMs and the underlying atomic rearrangement processes.

### 1.3 Discrepancies Regarding the Structure of Amorphous GeTe

This has motivated many previous studies. Nevertheless, these studies have not resulted in an unambiguous scenario. Previous studies reveal that there is a systematical deviation between a‐GeTe produced by melt‐quenching and a‐GeTe prepared by magnetron sputter‐ or vapor‐deposition. For the former case^[^ ^5^, ^21^, ^22^, ^23^, ^24^, ^25^ ^]^ a rather octahedral coordination is reported, in contrast to the latter case,^[^ ^26^, ^27^, ^28^, ^29^, ^30^ ^]^ where a predominantly tetrahedral coordination is found. Thus, the local amorphous structure of GeTe is considered to be strongly dependent on the preparation conditions.^[^ ^14^, ^31^, ^32^ ^]^ As evidenced via simulations of both the melt‐quench process and the deposition process, the amorphous atomic structures are also considerably different in the related PCM Ge_2_Sb_2_Te_5_. Again an increased fraction of tetrahedral sites is found in the case of sputter deposition.^[^ ^33^ ^]^ This implies that preparation conditions are seemingly more advantageous for the formation of tetrahedral environments for amorphous materials produced by sputter deposition.^[^ ^33^, ^34^ ^]^ The origin for the discrepancies may be elucidated by inspecting the liquid phase from which melt‐quenched a‐GeTe is produced. The SRO of liquid GeTe shows an octahedral character.^[^ ^35^, ^36^, ^37^ ^]^ At 900 K or above (from where the melt‐quench process is initialized^[^ ^5^, ^21^, ^22^ ^]^), the order parameter *r*_2_/*r*_1_ = 1.42(2)^[^ ^36^ ^]^ is close to the octahedral limit of *r*_2_/*r*_1_ = 1.41. It is therefore reasonable that many octahedral remnants persist during cool‐down. Owing to the fast melt‐quench from the liquid phase, especially in the case of simulations that use extremely high quench rates (*e. g*. ^[^ ^21^ ^]^ ϑ = 33.3 · 10^12^ K/s), it is not surprising that the average structure of the quenched solid is more similar to the liquid phase.

Also, the substrate temperature during the deposition process was reported to have a significant effect on the amorphous structure: A reduction of the substrate temperature results in a more octahedral‐like coordination compared to the structure produced by evaporation at room temperature.^[^ ^38^ ^]^

In addition to the influence of the preparation method on the atomic structure, discrepancies between experimental results and DFT simulations of disordered GeTe can arise. It has been observed that simulations of amorphous GeTe are generally prone to systematically increased bond lengths between Ge and Te sites.^[^ ^39^, ^40^ ^]^ Since the tetrahedral motifs are considered to have shorter bond lengths, the systematically increased bond length in simulations also contributes to a tendency to overestimate the actual amount of octahedral motifs.^[^ ^14^, ^33^, ^41^ ^]^

One of the well‐known structural peculiarities of crystalline GeTe is the Peierls distortion.^[^ ^42^ ^]^ These Peierls distortions are frequently encountered in heavy chalcogenides such as GeTe, Sb_2_Te_3_, or Bi_2_Se_3_, which employ a bonding mechanism, which differs from ordinary covalent, metallic, or ionic bonding. This bonding mechanisms has been coined metavalent.^[^ ^43^, ^44^ ^]^ It is characterized by an unconventional property portfolio,^[^ ^45^ ^]^ an unusual bond rupture,^[^ ^46^ ^]^ and distinct quantum chemical bonding descriptors.^[^ ^47^ ^]^ The bonding is characterized by a competition between electron localization and electron deloclization. Such solids often show Peierls distortion that raises the bandgap and increases the electron localization.^[^ ^22^, ^48^, ^49^ ^]^ Starting from the perfect octahedral GeTe structure (NaCl), the Peierls distortion is manifested in a shift of one of the (Ge or Te) sublattices in the (1 1 1) direction. That is, instead of each octahedral site having six equally distant nearest neighbors, they divide into three long bonds and three short bonds. Rephrased, the first coordination shell (*r*_1_) is split into three short (*r*_S_) and three long (*r*_L_) bonds in an octahedral atomic environment. A related Peierls‐like distortion (PD‐like) with even larger amplitude is often observed in the amorphous phase of GeTe.^[^ ^22^, ^50^ ^]^ In the amorphous phase this distortion can easily lead to the misinterpretation of octahedral units as tetrahedral sites in GeTe. Various methods to distinguish between tetrahedral and octahedral arrangements of an atomic configuration in GeTe exist, which differ in the specification of a cutoff distance for the first coordination shell. A structural motif usually is defined to be tetrahedral or octahedral based on the atoms that are considered to be part of this coordination shell. From these atoms within the cutoff distance the coordination number, the bond angles and/or an order parameter are deduced to discern tetrahedral from octahedral motifs. However, as a result of the large distortion in a‐GeTe the long bond distance *r*_L_ is close to the bond distance of the second coordination shell *r*_2_. This is problematic for the specification of a convenient cutoff distance for the first coordination shell.

In total, there are several reasons that can lead to the discrepancies about the tetrahedral fraction in a‐GeTe: The different preparation conditions, the systematic deviation of the Ge‐Te bond length in simulations and the difficulty to discriminate between tetrahedral and octahedral motifs due to PD‐like arrangements in a‐GeTe. Lastly, using one of the several different approaches for estimating the tetrahedral fraction may lead to variations in the final result as well.

In this study changes to the amorphous phase upon structural relaxation of GeTe produced by magnetron sputter deposition is examined experimentally by ePDF analysis via TEM measurements and corroborative RMC simulations. Also another approach for the determination of the tetrahedral to octahedral/PD‐like fraction using the atomic distance distributions of nearest‐neighbors is applied.

## 2 Results and Discussion

### 2.1 Correction for Inelastic Scattering

A general introduction and definitions of the usual quantities like the structure function *S*(*q*), the reduced structure function ϕ(*q*) and the pair distribution function *G*(*r*) can be found in the Section SI (Supporting Information). An elaborate correction procedure is required to correct the structure function *S*(*q*) for inelastic scattering, otherwise a significant distortion in the *S*(*q*)‐data would lead to unphysical features in the resulting PDF curves. Additionally, correcting the distortion in *S*(*q*) enables the extraction of the order parameter *S*(*q*_2_)/*S*(*q*_1_). However, not only inelastic scattering but also multiple scattering can ‐ to a lesser extent ‐ cause low frequency distortions in *S*(*q*). In addition, it can also affect the peak shape to a minor extent. Due to the generally broad amorphous *S*(*q*)‐maxima an impact on the peak shape due to multiple scattering can be considered negligible in the final PDF.^[^ ^7^ ^]^ Moreover, with a reduced film thickness of only 30 nm, it was ascertained that the principal reason for the distortion in *S*(*q*) was not multiple scattering but inelastic scattering.

Inspired by other literature^[^ ^7^ ^]^ where a polynomial fit is employed to remove the parasitic distortion of the structure function, a similar but data‐rooted approach is presented in the following.

In contrast to the polynomial fit that is simply subtracted from the uncorrected structure function, the correction technique elaborated here will enable an analysis of the uncertainties on the peak heights and positions. This justifies a meaningful and reliable discussion of the results of the order parameters upon relaxation. However, this type of data modification to correct for inelastic scattering distortions has the disadvantage that a quantitative analysis (*i. e*., the determination of coordination numbers) is questionable.^[^ ^7^ ^]^ Therefore, the impact of such a correction on a quantitative PDF analysis of amorphous SiO_2_ via RMC methods is envisaged prior to the analysis of GeTe to ensure the viability of the approach chosen.

**Figure 1:**
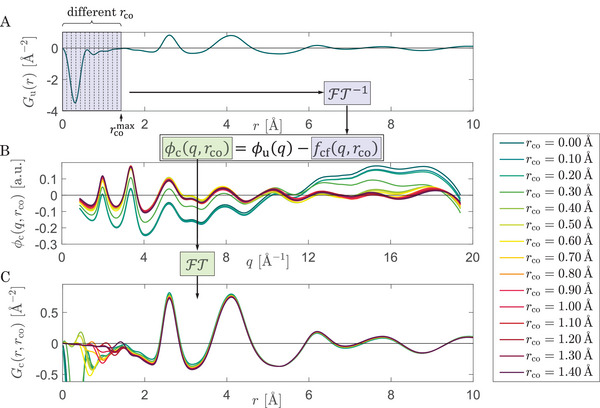
Overview of the correction procedure. A) An uncorrected pair distribution function (PDF) is shown (*cf*. deep valley in shaded area is unphysical). Different correction functions can be obtained by Fourier transforming the untreated PDF inversely in the region from *r* = 0 to different *r*_co_. B) By subtracting the correction functions (defined by different *r*_co_) from the uncorrected reduced structure function, one yields a set of corrected reduced structure functions ϕ_c_(*q*, *r*_co_). One can observe a clear decline in the distortion by increasing *r*_co_. C) From the set of corrected reduced structure functions a set of corrected PDFs *G*_c_(*r*, *r*_co_) can be calculated by Fourier transformation. Similar to the corrected reduced structure function, the corrected PDFs show a continuous decline of their unphysical feature, *i. e*. the valley in the low‐*r* regime.

The correction procedure is illustrated in **Figure** **1**. It is performed using the reduced structure function ϕ(*q*) adopted from eRDF Analyser.^[^ ^51^ ^]^ ϕ(*q*) is directly linked to the PDF *G*(*r*) via its Fourier transform and to the structure function *S*(*q*) via *S*(*q*) = ϕ(*q*)/*q* + 1. For a brief introduction and definitions used within the program refer to Section SI A (Supporting Information). A correction function *f*_cf_(*q*) must be found to be subtracted from the uncorrected reduced structure function ϕ_u_(*q*), resulting in the corrected reduced structure function

(1) $$\phi_{c}\left(q\right)=\phi_{u}\left(q\right)-f_{\mathrm{cf}}\left(q\right)$$

The low frequency distortion owing to inelastic scattering in ϕ_u_(*q*) is also observable in its Fourier transform, *i. e*., in the uncorrected PDF *G*_u_(*r*) (*cf*. shaded area in Figure 1 A). Normally, a straight line with negative slope proportional to the number density of the investigated material is expected in the *r*‐range prior to the nearest‐neighbor peak. In our case, below *r* < 1.5 Å an unphysical valley describes the undesired distortion. Accordingly, the inverse Fourier transform of this limited part of *G*_u_(*r*) is a good approach to establish the correction function:

(2) $$f_{\mathrm{cf}}\left(q,r_{\mathrm{co}}\right)={\mathrm{FT}}^{-1}\left[\Theta \left(r\right)G_{u}\left(r\right)\Theta \left(r_{\mathrm{co}}-r\right)\right]$$

Θ(*r*) denotes the Heaviside‐function ensuring that only the part between *r* = 0 and *r* = *r*_co_ is transformed. *r*_co_ is hence the upper cutoff value for the correction. FT[·] and ${\mathrm{FT}}^{-1}\left[\;\cdot \;\right]$ denote the sine‐wave Fourier‐transform as is used to compute *G*(*r*) and the inverse operation, respectively. A pair distribution function that is corrected with the correction cutoff distance *r*_co_ is then found from

(3) $$G\left(r,r_{\mathrm{co}}\right)=\mathrm{FT}\left[\phi_{c}\left(q,r_{\mathrm{co}}\right)\right]=\mathrm{FT}\left[\phi_{u}\left(q\right)-f_{\mathrm{cf}}\left(q,r_{\mathrm{co}}\right)\right]$$

The main advantage of this technique is the possibility to evaluate the uncertainties induced in the calculated PDF and the structure function as a function of the cutoff distance *r*_co_. Via a step‐wise increase from *r*_co_ = 0 Å (*i. e*., no correction) to $r_{\mathrm{co}}=r_{\mathrm{co}}^{\max}$ (maximum correction), any changes due to the correction can be analyzed in terms of ϕ(*q*) and *G*(*r*) (*cf*. Figure 1). The peak positions and the resulting order parameters evolve with increasing *r*_co_ which can be resolved and evaluated. This helps estimating a total uncertainty on the quantities extracted.

To find an appropriate range of *r*_co_, such that the correction is large enough to fully remove the distortion from the structure function but simultaneously ensuring not to depress any physical peaks in the resulting PDF, the correction degree *C*_D_(*r*_co_) is introduced as a measure of the difference between uncorrected and corrected structure function. A cutoff distance of *r*_co_ = 1.0 Å is chosen (*cf*. Sec. S‐I E in the SI), which is far below the expected atomic distances in GeTe (≈2.6 Å). A variation of ± 0.3 Å shall account for the uncertainty resulting from the correction procedure. Every quantity is averaged over this correction range *r*_co_ ∈ *R*_co_, where *R*_co_ is defined as the set

(4) $$R_{\mathrm{co}}:=\left\{0.7,0.8,0.9,1.0,1.1,1.2,1.3\right\}\;Å$$

That is, the two functions that are consolidated for the analysis are generated by:

(5) $$S\left(q\right)=\mathrm{mean}\{\;S\left(q,r_{\mathrm{co}}\right);\;r_{\mathrm{co}}\in R_{\mathrm{co}}\;\}$$

and

(6) $$G\left(r\right)=\mathrm{mean}\{\;G\left(r,r_{\mathrm{co}}\right);\;r_{\mathrm{co}}\in R_{\mathrm{co}}\;\}$$

### 2.2 Fitting of Order Parameters *S*(*q*_2_)/*S*(*q*_1_) and *r*_2_/*r*_1_

It is important to obtain reliable data for *G*(*r*) in order to derive conclusions about structural changes in a material of interest. That is why in the previous section a new correction scheme was developed and recapitulated (more details *cf*. Section SI, Supporting Information). Given that reliable *G*(*r*) data can now be obtained from electron diffractometry in TEM, structural parameters have to be defined that clearly reveal structural changes. Here, the two order parameters *S*(*q*_2_)/*S*(*q*_1_) and *r*_2_/*r*_1_ are employed. *S*(*q*_2_)/*S*(*q*_1_) relates the peak heights of the first two maxima in the structure function from which the predominant structural motifs in a disordered material can be concluded^[^ ^13^ ^]^:

(7) $$\frac{S(q_{2})}{S(q_{1})}\left\{\begin{array}{c} <1\;\Rightarrow \mathrm{octahedral}\\ >1\;\Rightarrow \mathrm{tetrahedral} \end{array}\right.$$

The quantity *r*_2_/*r*_1_ is defined by the ratio between the average second nearest‐neighbor to first nearest‐neighbor distance in *G*(*r*). Using geometric arguments this ratio can be used to judge whether the atomic arrangements are predominantly octahedral or tetrahedral^[^ ^13^, ^52^, ^53^ ^]^:

(8) $$\frac{r_{2}}{r_{1}}=\left\{\begin{array}{cc} \sqrt{2} & \approx 1.41\;\Rightarrow \mathrm{octahedral}\\ \frac{4}{\sqrt{6}} & \approx 1.63\;\Rightarrow \mathrm{tetrahedral} \end{array}\right.$$

The order parameters have been used simultaneously to investigate glassy or liquid systems, which are either predominantly octahedral or tetrahedral. Especially *S*(*q*_2_)/*S*(*q*_1_) for eleven chalcogenide compounds has been observed to be clearly below unity (*S*(*q*_2_)/*S*(*q*_1_) = 0.66…0.88) for mostly octahedral systems with *r*_2_/*r*_1_ = 1.37…1.49 or clearly above unity (*S*(*q*_2_)/*S*(*q*_1_) = 1.25…1.31) for mostly tetrahedral systems with *r*_2_/*r*_1_ = 1.60…1.65.^[^ ^13^ ^]^ However, these order parameters have also been used independently, *e. g*., in ref. [52], where a pressure induced transition from tetrahedral to octahedral has clearly been shown using *r*_2_/*r*_1_ for Cu halides. While *r*_2_/*r*_1_ unambiguously classifies the compounds into tetrahedral or octahedral at the end points of the transition, *S*(*q*_2_)/*S*(*q*_1_) is not used as a parameter as the situation seems to be not that clear. Concluding, the order parameters can be used to verify trends but shall not be used as an absolute measure.

As mentioned in Section 1, the Peierls‐like distortion (PD‐like motifs) can be found in amorphous GeTe, as well as a large variety of different geometries and bond lengths. Therefore, difficulties in the interpretation of these simple order parameters can arise in our case, which is why complementary RMC simulations are conducted as well. The order parameters *S*(*q*_2_)/*S*(*q*_1_) and *r*_2_/*r*_1_ have to be extracted from *S*(*q*) and *G*(*r*). For this purpose, Gaussian fits are conducted at the first two principal peaks of *S*(*q*) and *G*(*r*), respectively. *S*(*q*_*i*_) and *r*_*i*_ can then be obtained from the Gaussian peak maximum height and peak position. Fitting uncertainties σ_fit_ are estimated as described in the SI (Section SI F, Supporting Information). But *r*_*i*_ and *S*(*q*_*i*_) shall not simply be extracted from fitting the averaged functions (Equations 6 and 5) since the uncertainty due to the correction can be estimated more accurately if one proceeds as follows: The quantities are computed for each of the step‐wise (*r*_co_ ∈ {0, 0.1, …, 1.4}) corrected curves individually. By doing so, an evolution upon correction is documented and the influence of the correction procedure on the quantities can be understood (*cf*. Section SI G, Supporting Information).

All the required values for *r*_2_/*r*_1_ and *S*(*q*_2_)/*S*(*q*_1_) are averaged over the reasonably chosen set of *R*_co_. The uncertainty σ_c, *Q*_ of a quantity *Q* due to the correction is calculated by the standard deviation:

(9) $$\begin{array}{cc} Q & =\mathrm{mean}\{\;Q\left(r_{\mathrm{co}}\right);\;r_{\mathrm{co}}\in R_{\mathrm{co}}\;\} \end{array}$$

(10) $$\begin{array}{cc} \sigma_{c,Q} & =\mathrm{std}\{\;Q\left(r_{\mathrm{co}}\right);\;r_{\mathrm{co}}\in R_{\mathrm{co}}\;\} \end{array}$$

where *Q* ∈ {*r*_1_, *r*_2_, *S*(*q*_1_), *S*(*q*_2_)} stands for the quantity of interest. It shall be mentioned that computing the standard deviation was done without dividing by $\sqrt{N}$ such that an artificial decrease of the uncertainty by including more *r*_co_ data points in between the existing ones is not possible.

The total uncertainty on a quantity *Q* is finally given by:

(11) $$\sigma_{Q}=\sqrt{{\left({\hat{\sigma }}_{\mathrm{fit},Q}\right)}^{2}+{\left(\sigma_{c,Q}\right)}^{2}}$$

where

(12) $${\hat{\sigma }}_{\mathrm{fit},Q}=\max\left\{\sigma_{\mathrm{fit},Q}\left(r_{\mathrm{co}}\right);\;r_{\mathrm{co}}\in R_{\mathrm{co}}\right\}$$

is the largest fitting error that was determined for the quantity *Q* in the set of correction ranges *R*_co_. An error due to measurement uncertainties is also considered (*cf*. Section SI H, Supporting Information).

### 2.3 Validity of the ePDF Method and the Correction Applied

To test the modified ePDF analysis technique including the correction scheme developed several approaches have been chosen. First, measurements on amorphous C, Si, SiO_2_, Si_3_N_4_ thin film samples have been conducted and were analysed. These materials are so‐called Zachariasen glasses,^[^ ^54^ ^]^ which means that the coordination and bond length in the crystalline and the glassy (amorphous) state are equal. This close similarity of the short‐range order has been attributed by Zachariasen to the similarity of bonding in both configurations. As the atomic positions and thus the bond lengths in a crystal are measured with great precision, the atomic distances from the amorphous phases of C, Si, SiO_2_ and Si_3_N_4_ are compared to their crystalline counterpart to judge the reliability of our approach.

Second, a crystalline Ge_2_Sb_2_Te_5_ film is measured with our technique and its PDF is also compared to the expected crystal structure, to test the limits of the technique regarding crystalline systems. Third, the data measured on amorphous SiO_2_ is used for a structural model based on an RMC simulation and this model, including coordination numbers and bond angles, is compared to other RMC models obtained from X‐ray or neutron scattering data. Lastly, our *S*(*q*) and *G*(*r*) data for amorphous GeTe has also been compared to earlier studies about the structure of amorphous GeTe. In the following, the first three testing methods are discussed. The comparison of *S*(*q*) and *G*(*r*) of amorphous GeTe from former studies and from the current work can be found in the Supporting Information (Section SIII, Supporting Information).

#### 2.3.1 Qualitative Analysis ‐ Peak Positions

**Figure 2:**
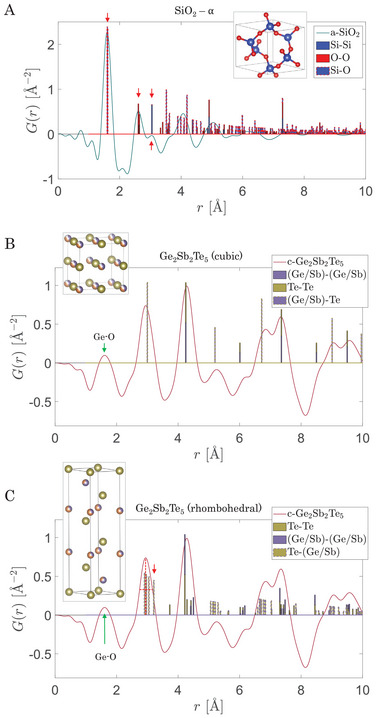
Comparison of our measured data (solid lines) to crystal structures (bars). The unit cell and name of the corresponding model which the data is compared to is shown in the graphs. By using CIF2PDF the crystalline distances have been calculated from the crystallographic information files listed in the SI, Table S2 (Supporting Information). A) For SiO_2_ we expect the same short‐range order of the crystal and the glass. Hence, the good agreement of the first maxima of *G*(*r*) for the glass (solid blue line) with the crystalline peaks of the PDF validate the measurement and analysis procedure developed. B,C) PDF of crystalline Ge_2_Sb_2_Te_5_ (solid red line, identical in B and C) compared to the cubic (octahedral) and the rhombohedral crystalline phase, respectively. From these two comparisons it can be inferred that the crystalline Ge_2_Sb_2_Te_5_ sample is not in the purely cubic phase but a Peierls instability occurs, observable from the asymmetry in the first major maximum (red arrow).

For the Zachariasen glasses C, Si, SiO_2_ and Si_3_N_4_ a match between the first two maxima *r*_1_ and *r*_2_ in the experimental amorphous PDF and the two shortest and most prominent occurring crystalline distances is expected. The amorphous SRO of SiO_2_ closely resembles the SRO of the α‐SiO_2_ crystal structure. The largest crystalline peaks coincide with the amorphous maxima up to the right shoulder of the second coordination shell, after which the glass loses structural coherence (cf. **Figure** **2** a). For the other Zachariasen glasses C, Si and Si_3_N_4_ an equally good matching is found (*cf*. Section SII, Supporting Information ). To further investigate the precision beyond the first two peaks, a crystalline Ge_2_Sb_2_Te_5_ sample (crystallized by annealing for 1 h at 150 °C) was investigated and compared to the crystalline models as well (Figure 2 b,c). The small pre‐peak at ≈ 1.5…2.0 Å is traced back to surface oxidation of the film (it was not kept under argon atmosphere in contrast to the GeTe samples discussed below) resulting in Ge‐O bonds in this *r*‐range. For peaks above 2 Å, the similarity of the investigated crystalline Ge_2_Sb_2_Te_5_ film (red solid line) to the calculated cubic crystal structure (bars) becomes apparent, as all measured maxima find a counterpart in the cubic phase of Ge_2_Sb_2_Te_5_ as Figure 2 b demonstrates. Nevertheless, minor discrepancies in the perfect alignment of the peaks occur, in particular in the ascending branches at ≈ 6 Å or ≈ 6.8 Å. Also, the first crystalline peak cannot perfectly describe the first measured maximum with its slight asymmetry. However, due to the Peierls instability of the Te atoms at room temperature in crystalline Ge_2_Sb_2_Te_5_, a distortion in the cubic lattice is introduced.^[^ ^55^ ^]^ The rhombohedral crystal phase (*cf*. bars in Figure 2 c) is characterized by a Peierls distortion, manifested in multiple peak splittings in the crystalline PDF. The distorted crystal structure can describe the asymmetric feature in the first maximum (red arrow). Overall, the measured crystalline Ge_2_Sb_2_Te_5_ film can be described very well by the existing models. Especially the minor distortion due to the Peierls instability could be identified correctly. Hence, the method used to determine the SRO from SAED of amorphous and crystalline samples yields qualitatively reasonable results up to at least 6 Å.

#### 2.3.2 Quantitative Analysis ‐ Coordination Number and Bond Angle Distribution

**Figure 3:**
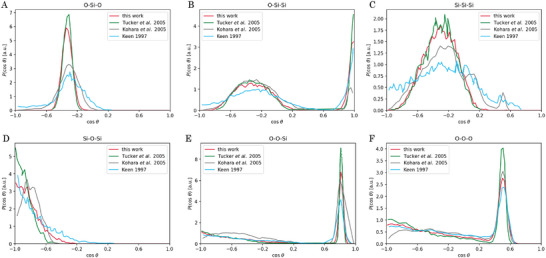
Bond angle distributions of SiO_2_. Red curves show the result of the current study with the cutoff distances for Si‐O (2.1 Å), O‐O (3.2 Å) and Si‐Si (3.7 Å) defined by the minimum of each partial PDF. The other curves are replottted from Tucker^[^ ^56^ ^]^ et al., Kohara^[^ ^57^ ^]^ et al. and Keen^[^ ^58^ ^]^ et al. An excellent agreement with the high‐quality simulations based on neutron diffraction data from Tucker et al. is found.

For SiO_2_ an RMC simulation is conducted to estimate the validity of a quantitative analysis via coordination numbers (CNs) and bond angle distributions (BADs). Simulation details can be found in the Supporting Information (Section SV, Supporting Information). In the RMC simulation, real‐space and reciprocal‐space data have been fitted simultaneously and the resulting partial PDFs are in good agreement with existing models for amorphous SiO_2_ (*cf*. Figure S10, Supporting Information). The principal peaks of the partial PDFs are to a small extent broader compared to simulations based on neutron‐ or X‐ray data. That is attributed to the smaller *q*‐range being just above 19 Å^−1^ for the electron data. Calculating the coordination number of O atoms around Si centers from the partial PDFs a value of 3.94 is obtained, which is reasonably close to the expected four‐fold coordination. Based on the bond angle distributions (BADs) of the SiO_2_ structure the simulated configuration represents the features of existing amorphous SiO_2_ models very well (*cf*. **Figure** **3**a–f) and proves the quality of the model presented here. In conclusion, a very good agreement between the RMC simulations based on electron diffraction data and X‐ray/neutron data is obtained and reliable results regarding a quantitative analysis can be deduced by the method applied.

### 2.4 Structural Relaxation in a‐GeTe

#### 2.4.1 Order Parameters *S*(*q*_2_)/*S*(*q*_1_) and *r*_2_/*r*_1_

We now turn our attention to the structural changes in amorphous GeTe. The resulting *S*(*q*) and *G*(*r*) curves for differently treated samples are given in Figures S8 and S9 (Supporting Information), revealing obvious changes upon thermal treatment.

**Figure 4:**
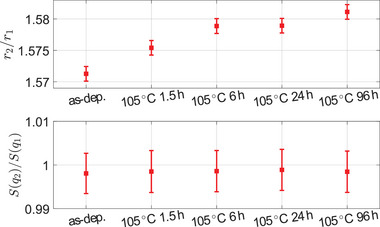
Order parameters *r*_2_/*r*_1_ and *S*(*q*_2_)/*S*(*q*_1_) upon structural relaxation. While a continuous increase of *r*_2_/*r*_1_, potentially indicative of an increase in tetrahedral coordination, can be observed upon aging, *S*(*q*_2_)/*S*(*q*_1_) stays constant instead of increasing towards the nominally tetrahedral limit. This behavior may be explained by the structural motifs in a‐GeTe being heavily distorted octahedral. This presumably also results in the *S*(*q*_2_)/*S*(*q*_1_)‐ratio to be close to the transition range of unity. More insights shall be obtained by analyzing the RMC simulations.

The extrema of *S*(*q*) in the range of *q* > 6 Å^−1^ shift toward larger values, which translates to a shift of the first peak in *G*(*r*) from *r* > 2.61 Å to shorter atomic distances. The order parameters *r*_2_/*r*_1_ and *S*(*q*_2_)/*S*(*q*_1_) from the experimental data are shown in **Figure** **4**. While *r*_2_/*r*_1_ is clearly increasing from about 1.57 to 1.58 upon structural relaxation of the glassy phase, no change in *S*(*q*_2_)/*S*(*q*_1_) can be observed. The values for *r*_2_/*r*_1_ are closer to the limit of tetrahedral (*r*_2_/*r*_1_ ≈ 1.63) coordination (*vs*. octahedral *r*_2_/*r*_1_ ≈ 1.41). However, *S*(*q*_2_)/*S*(*q*_1_) is found to be in an intermediate range (*S*(*q*_2_)/*S*(*q*_1_) = (0.998…0.999) ± 0.005) around unity, which should divide the cases of rather tetrahedral (*S*(*q*_2_)/*S*(*q*_1_) > 1) and octahedral (*S*(*q*_2_)/*S*(*q*_1_) < 1) coordination. The rather invariant order parameter *S*(*q*_2_)/*S*(*q*_1_) upon annealing indicates that the structural changes are more complex than just an increase in tetrahedral coordination upon annealing as might be inferred from the increase of *r*_2_/*r*_1_ alone. This complex structural relation of amorphous GeTe is mirrored in reported values in the literature: Studies^[^ ^28^, ^29^, ^39^, ^59^ ^]^ of amorphous GeTe produced by sputter‐ or vapor‐deposition report *S*(*q*_2_)/*S*(*q*_1_)‐ratios in the range of 0.79 to 0.97 (0.89 on average).^[^ ^14^ ^]^ These variations may also be a result of different sample treatments; specifically, different substrate temperatures (room temperature vs 70 °C) and sputter powers during deposition (20 W in the current study vs 40 W vs 100 W) and varying mechanical stresses (or no mechanical treatment as in the current study) to scratch off the sputtered film to produce the powder samples. An increase in *r*_2_/*r*_1_ toward 1.63 accompanied by an increase of *S*(*q*_2_)/*S*(*q*_1_) above unity is generally associated with an increase of tetrahedral coordination.^[^ ^13^ ^]^ However, the correlation between *S*(*q*_2_)/*S*(*q*_1_) and *r*_2_/*r*_1_ has only been investigated for truly octahedral (*S*(*q*_2_)/*S*(*q*_1_) < 0.88; *r*_2_/*r*_1_ < 1.49) or truly tetrahedral (*S*(*q*_2_)/*S*(*q*_1_) > 1.25; *r*_2_/*r*_1_ > 1.60) disordered systems in the referenced literature. Thus, the case of a‐GeTe where heavily distorted octahedral motifs occur has not been completely covered. We hence have to analyze further data. Fortunately, we can evaluate the ϕ(*q*) and *G*(*r*) curves and the changes therein with the RMC methods developed in refs. [60, 61].

#### 2.4.2 Coordination Numbers

**Figure 5:**
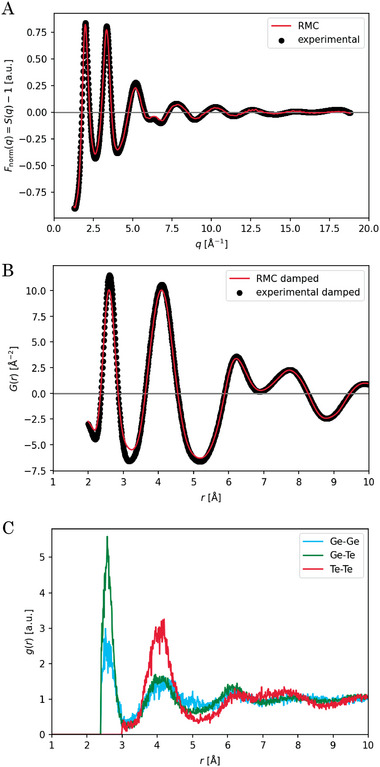
Exemplary RMC fits of the as‐deposited GeTe sample. A) Structure function *F*_norm_(*q*) = *S*(*q*) − 1 as defined in the RMCProfile manual.^[^ ^62^ ^]^ Please note that the definitions of *S*(*q*) and *G*(*r*) have been adopted from the original publication of the eRDF Analyser software^[^ ^51^ ^]^ and are not according to those in the RMCProfile manual. Up to transformation pre‐factors the correspondence is as follows: *S*(*q*)→ *S*_norm_(*Q*) and *G*(*r*)→ *D*(*r*). B) Pair distribution function *G*(*r*) computed from the structure function shown in A with Lorch damping applied. C) Partial pair correlation functions. The fits of *F*_norm_(*q*) and *G*(*r*) are very satisfactory and representative of the quality of all RMC fits performed in this study.

**Figure** **5** exemplary depicts the fit results for one of the RMC runs of the as‐deposited sample. A good agreement between simulation and experimental data was achieved.

The Ge coordination number (CN) yields values that are compatible with the range of former works' results and do not change significantly upon relaxation (*cf*. Table S1, Supporting Information): CN(Ge‐Ge) ≈ 1.44, CN(Ge‐Te) = CN(Te‐Ge) ≈ 2.50, N_tot_(Ge) =CN(Ge‐Ge) +CN(Ge‐Te) ≈ 3.94. Only for the Ge‐Ge coordination a minor decrease upon annealing becomes obvious, *cf*. Figure S11 (Supporting Information).

The fraction of homopolar Ge bonds (*i. e*., Ge‐Ge bonds) does not show a strong trend and is around 23 % and 24 % for a bond length cutoff at 3.0  and 3.2 Å, respectively.

#### 2.4.3 Structural Motifs

To gain a deep understanding of changes of tetrahedral Ge sites upon annealing, here the tetrahedral fraction of Ge‐centered motifs is determined by three different methods: First, the order parameter $p=1-\frac{3}{8}\sum_{i>k}{\left(\frac{1}{3}+\cos\theta_{ijk}\right)}^{2}$, where θ_*ijk*_ denotes the angle spanned by the central atom *j* with the two neighboring atoms *i* and *k*.^[^ ^63^ ^]^ Out of all Ge centers, those that are fourfold coordinated and fulfill 0.8 < *p* < 1.0 provide a measure for the fraction of tetrahedral Ge sites.^[^ ^37^, ^64^, ^65^ ^]^ For this analysis, a fourfold motif is defined by a central atom with exactly four adjacent atoms within the cutoff range (varied between 3.0 and 3.2 Å). Second, the order parameter *d*_4_/*d*_0_, where *d*_4_ is the distance of the fourth nearest neighbor to the central Ge atom and *d*_0_ is the reference bond distance by which *d*_4_ is normalized. The reference distance *d*_0_ is set to 2.65 Å for a mixed (Te‐containing) motif and to 2.50 Å in case of a motif consisting purely of Ge atoms (since the Ge‐Ge bonds are shorter in general; *d*_GeGe_ = 2.45 Å in elemental tetrahedral Ge, *cf*. data in Table S2, Supporting Information). The tetrahedral share using that parameter is given by the fraction of central Ge atoms that fulfill 1.0 < *d*_4_/*d*_0_ < 1.1.^[^ ^22^ ^]^ The argument for that order parameter is the difference in bond distance for tetrahedral (shorter) and octahedral (longer) bonding in a‐GeTe.

**Figure 6:**
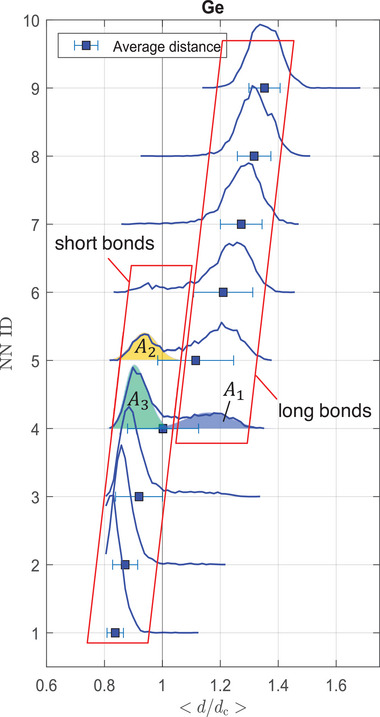
Distance distribution of nearest neighbors (DDNN) for Ge‐centers and how a measure for the fraction of Ge‐centered motifs can be inferred. For the first nine nearest neighbors (NN ID = 1…9) the probability of their distance to the central Ge atom are shown as blue curves. The distances *d* are normalized to the crystalline bond length of GeTe with *d*_*c*_ = 3.00605 Å. A bimodal distribution becomes apparent with short and long bonds, particularly on the fourth ID reflecting the coexistence of fourfold tetrahedral and threefold PD‐like Ge motifs. Double Gaussian fits to the 4th and 5th NN can be used to obtain a measure of the distinct motifs, *cf*. Section SVI (Supporting Information).

**Figure 7:**
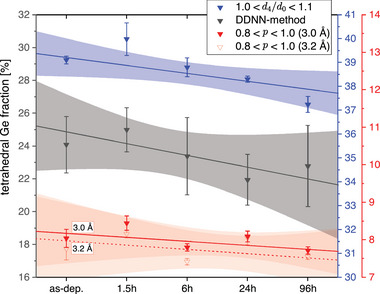
Calculated fraction of tetrahedral Ge‐centered motifs upon annealing at 105 °C based on different methods. The total fraction of the three different methods deviate significantly from each other, indicative of the difficulty to determine a good absolute measure of tetrahedricity in a‐GeTe. The consistently declining linear fits for the concentration of tetrahedral Ge‐motifs upon aging are indicative for structural relaxation away from tetrahedral Ge sites.

The third method introduced in this work does not require a cutoff distance or range of order parameters, which is a clear advantage. It is solely based on the individual distance distributions of nearest neighbors (DDNN, *cf*. **Figure** **6**), *i. e*., the probability *P*(*d*_*i*_) that the *i*th NN is a certain distance apart from the central atom. This method yields unique fingerprints for ideally tetrahedral, octahedral and heavily Peierls distorted/pyramidal structures (*cf*. Figure S14, Supporting Information). That means that in particular tetrahedral structures can be discerned from heavily PD‐like structures. This is advantageous over the angular description of the motif. The concept is introduced in the Supporting Information in more detail (*cf*. Section SVI, Supporting Information) and shall be compared to the other two methods' results in the following. Examining the measures (*cf*. **Figure** **7**) reveals that the results for the tetrahedral Ge fractions determined by the different methods vary substantially between ≈8% and ≈40%.

These differences are striking. They are attributed to the different degrees of selectivity for a motif. On the one hand‐side, the selection rule for a tetrahedral motif based on the order parameter *d*_4_/*d*_0_ is rather loose since no bond angles around the Ge center are considered and solely the bond distances are used as an argument in that case. This rather loose definition probably leads to an increased amount of tetrahedral Ge centers that are counted (≈40%). On the other hand‐side, the order parameter *p* takes into account the specific bond angles of the nearest neighbors. In fact, this is the most rigorous and stringent selection rule, which also results in the lowest fraction of tetrahedral Ge (≈8%). However, in that case a cutoff distance must be defined to discriminate between the first and second coordination shell. Due to the strong Peierls‐like distortions in amorphous GeTe the long bond distance of the first coordination shell is close to that of the second coordination shell. Deciding where to set a cutoff manually is difficult and might be prone to bias. Furthermore, a range for *p* needs to be defined. Usually, 0.8 < *p* < 1.0 is the range for tetrahedral motifs (*p* = 1.0 → perfect tetrahedron), but the lower border might be adjusted to a lower (*e. g*., 0.85 < *p* < 1) or higher (*e. g*., 0.75 < *p* < 1) degree of distortion that one wants to accept for a tetrahedral motif.

The DDNN method developed here serves as another independent measure, the selectivity of which represents a compromise between the loose definition of *d*_4_/*d*_0_ and the strict exclusion of motifs with neighbors beyond a certain cutoff distance in the definition of *p*. As expected, the outcome for the DDNN method (≈24%) lies inbetween the results from the two other order parameters.

We now turn our attention from the comparison of the absolute measures of the three methods to the relaxation behavior and, thus, observable trends in the data.

From the order parameter *p*, for which two cutoff distances (3.0 and 3.2 Å) are applied for the specification of fourfold Ge centers, a tetrahedral Ge fraction of about 8% is found. As the octahedral bonds in general tend to have an increased bond length compared to the tetrahedral bond length, it is reasonable that the fraction calculated with the shorter cutoff distance of 3.0 Å shows a slightly enhanced tetrahedral share. Decreasing the cutoff distance further toward 2.7 Å results in a drastic decrease in the tetrahedral fraction (*cf*. Figure S13, Supporting Information ), since most tetrahedral bonds seem to have a bond length between 2.65 and 3.0 Å  as argued for the order parameter *d*_4_/*d*_0_. For both cutoff distances (3.0  and 3.2 Å) a common trend upon structural relaxation toward lower tetrahedral coordination is found. A considerably larger tetrahedral Ge fraction is obtained by the order parameter *d*_4_/*d*_0_, which results in almost 40%. However, the same decreasing trend upon relaxation is observed.

**Figure 8:**
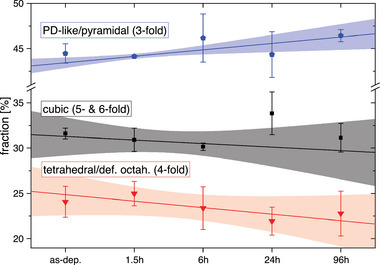
Fractions of different structural motifs computed by the distance distribution of nearest neighbors (DDNN‐method). Besides the decrease of fourfold and higher coordination a definite increase of heavily Peierls‐like distorted and pyramidal motifs (threefold coordination) becomes obvious.

The measure inferred from the DDNNs leads to an amount of about 24 % of tetrahedrally coordinated Ge atoms. Even though the uncertainty of these data points is comparably large (due to the tolerance of the double Gaussian fits, *cf*. Section SVI, Supporting Information ), a decreasing trend is obvious. Specifically, the consistent decrease of the tetrahedral Ge fraction in agreement with the other two methods' fits (*i. e*., using the order parameters *p* and *d*_4_/*d*_0_) is definite. This means that the suspicion of more complex structural rearrangement processes based on the non‐correlating behavior of the order parameters *S*(*q*_2_)/*S*(*q*_1_) and *r*_2_/*r*_1_ was correct. Here, the decrease in the tetrahedral Ge fraction is evident, while the *r*_2_/*r*_1_ would suggest otherwise. Due to the consistency of the methods to determine the tetrahedral Ge fraction, also the trends regarding the PD‐like/pyramidal fraction computed by the DDNN method are considered more reliable than the error bars suggest at first sight in **Figure** **8**. This figure shows the further results for the PD‐like/pyramidal and cubic fraction from the DDNN analysis. While the fourfold, fivefold and higher coordinated fraction is decreasing upon relaxation the threefold coordinated fraction (*i. e*., PD‐like/pyramidal) increases.

#### 2.4.4 Bond Angle Distribution

**Figure 9:**
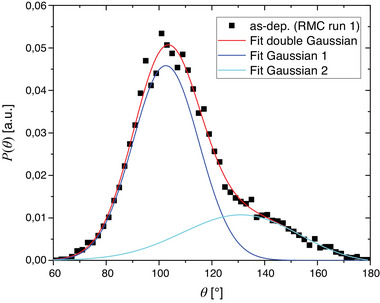
Exemplary Te‐Ge‐Te bond angle distribution of the RMC simulation run 1 of the as‐deposited phase. The cutoff distance was set to a large value of 3.2 Å to include as many angles as possible in the analysis, especially those where long bonds are involved. The distribution can be described well by a double Gaussian reflecting the effective bond angle potentials. Solid lines correspond to the fitted double Gaussians (red) and the constituting single Gaussians (blue and cyan).

**Figure 10:**
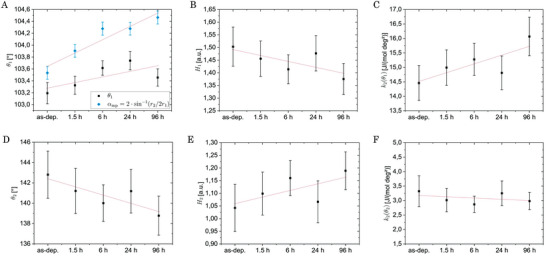
Results of the fits to the bond angle distributions (BADs) upon relaxation including a linear fit (red). A) Mean bond angle of the main Gaussian contribution θ_1_ (black) and most prominent bond angle α_mp_ calculated from the *r*_2_/*r*_1_‐ratio (blue). Both coherently show an increase toward larger bond angles. The offset of α_mp_ to larger angles is due to the fact that the geometries part of the secondary Gaussian with angles around 140 ° partly contribute to the measure of α_mp_. B) Normalized intensity of the main Gaussian contribution (θ_1_ ≈ 103 °) in the BADs. It decreases upon relaxation. C) Rigidity *k*_2_(θ_1_) of the effective bond angle potential of the main contribution. The increase in stiffness suggests an enhancement of the stability of the structures upon relaxation. D) Mean bond angle of the second Gaussian contribution θ_2_. The bond angle of the intermediate geometries show a decrease. E) Normalized intensity of the secondary Gaussian intermediate geometry contribution (θ_2_ ≈ 140 °) in the BADs. It increases upon relaxation. F) Rigidity *k*_2_(θ_2_) of the effective bond angle potential of the secondary contribution. The rigidity is much lower compared to the main contribution and shows a slight decreasing trend upon relaxation.

Further analysis of the Ge motifs is accomplished by computing the Te‐Ge‐Te bond angle distribution (BAD) for each RMC simulation run (four per annealing state). To reveal minor shifts in the BADs of different annealing states each BAD can be fitted to a Boltzmann probability distribution based on effective bond angle potentials $U_{\text{eff}}^{\left(i\right)}\left(\theta \right)$ ^[^ ^66^, ^67^ ^]^:

(13) $$\begin{array}{cc} P(\cos\theta ) & \propto \exp\left(-U_{\text{eff}}^{\left(i\right)}\left(\theta \right)/k_{B}T\right) \end{array}$$

(14) $$\begin{array}{cc} U_{\text{eff}}^{\left(i\right)}\left(\theta \right) & =\frac{k_{2}^{\left(i\right)}}{2}{\left(\theta -\theta_{i}\right)}^{2} \end{array}$$

where the Boltzmann constant is denoted by *k*_B_ and *T* is the temperature fixed at 300 K. θ_*i*_ is the effective potential minimum and $k_{2}^{\left(i\right)}$ the corresponding rigidity parameter, which gives a measure for the tolerance of a bond angle to deviate from the potential minimum. Thus, a large rigidity suggests that the tolerance is small and most motifs in the systems possess angles close to the mean value θ_*i*_. As the BADs show a bimodal distribution, a sum of two harmonic potentials is considered in the following. **Figure** **9** exemplarily shows the BAD of one RMC simulation run of the as‐deposited sample including the double Gaussian fit. The BADs feature one major contribution centered around ≈103° and a broad minor contribution at larger angles (≈120…160°). The BADs of the individual runs for one annealing state are fitted and from the results a weighted average and uncertainty is computed (based on the uncertainties returned by the fitting routine). Comparing the different annealing states reveals several observations:

The second broad contribution in the BAD is centered around 143° in the as‐deposited sample and shifts toward 139° upon annealing (*cf*. **Figure** **10**d). This shift of the contribution is accompanied by an increase of approximately 10 % in intensity (*cf*. Figure 10 e), which describes an enhancement in the occurrence of “intermediate geometries between regular tetrahedral and octahedral”.^[^ ^67^ ^]^ The rigidity of these motifs is not subject to pronounced changes upon annealing and is quite low (*cf*. Figure 10 f), which indicates that these structures are rather flexible and possess a variety of different bond angles.

The increased observation of motifs in the intermediate geometry range upon relaxation (while the mean angle decreases) can be traced back to a promotion of heavily Peierls‐like distorted (PD‐like) motifs as with the distortion not only the rectangular angles depart from 90° but also the 180° angles of opposite atoms decrease steadily toward the intermediate geometry range.

An increased occurrence of these intermediate geometries is accompanied by a decreased extent of resistance drift.^[^ ^67^ ^]^ Thus, the increase in intermediate (PD‐like) geometries observed here is in line with the observation of resistance drift observed in experiments.^[^ ^11^, ^12^ ^]^

An additional observation is that the main contribution is best described by an effective potential minimum at θ_1_ ≈ 103.2° that shifts toward larger angles upon structural relaxation (*cf*. Figure 10 a). That is a clear indication of the structure relaxing away from the perfectly octahedral bond angle of 90°, which was already observed in the increasing *r*_2_/*r*_1_‐ratio. The *r*_2_/*r*_1_‐ratio translates to the average most prominent bond angle α_mp_ = 2 · sin (*r*_2_/2*r*_1_), which follows the same trend as the main contribution of the BADs (blue data points Figure 10 a). That is, for the as‐deposited sample α_mp_ = 103.5° (*r*_2_/*r*_1_ = 1.571), while for the 96 h sample α_mp_ = 104.5° (*r*_2_/*r*_1_ = 1.581). The simultaneous decrease in tetrahedral motifs and the increase in Peierls‐like distorted motifs upon structural relaxation of amorphous GeTe may result in a rather constant order parameter *S*(*q*_2_)/*S*(*q*_1_) while the ratio *r*_2_/*r*_1_ increases.

The rigidity of the effective potential around the main contribution $k_{2}^{\left(\theta_{1}\right)}$ increases upon structural relaxation, *cf*. Figure 10 c. For amorphous Ge_*x*_Te_1 − *x*_ compounds different Ge fractions *x* caused a change in the stiffness $k_{2}^{\left(\theta_{1}\right)}$ of the main contribution in the Ge‐centered BAD. This change was accompanied by a change in the drift coefficient (describing the resistance drift quantitatively) as well. A larger stiffness $k_{2}^{\left(\theta_{1}\right)}$ can be associated with a lower drift coefficient, *i. e*., a slower relaxation.^[^ ^67^ ^]^ Thus, it is expected that upon resistance drift, which slows down exponentially with time, the stiffness $k_{2}^{\left(\theta_{1}\right)}$ must increase. This behavior of the stiffness is indeed what is observed here upon structural relaxation of amorphous GeTe.

#### 2.4.5 Classifying Results

In the review of Zhang et al. ^[^ ^68^ ^]^ previous literature is summarized to the view that upon structural relaxation Ge‐Ge bonds are broken and tetrahedral motifs transform to distorted octahedral motifs, which is in line with our findings. However, the literature cited to arrive to this view are all exclusively based on simulations such as molecular dynamics (MD) and do not refer to experimental data. Further reading in ref. [68] reveals that there is a discrepancy between work based on simulations and experimental data like the work published in ref. [32] where an increase in the tetrahedral fraction is concluded based on a shift of *r*_1_ toward lower distances and, thus, a larger fraction of homopolar Ge bonds. Based upon our experiments, we find the same trend for *r*_1_ as in ref. [32] which also points in the direction of an increase in the tetrahedral fraction (*r*_2_/*r*_1_ increase). Nevertheless, the here presented method in conjunction with RMC models reveals that the fraction of tetrahedral Ge motifs is decreasing, and distorted octahedral (PD‐like) motifs become more abundant while distortion continues to increase. Because of the similarity of the coordination shells in the case of amorphous GeTe the trend in *r*_2_/*r*_1_ gives a false (since incomplete) positive. With our newly developed method we are now able to experimentally identify the delicate changes that are taking place in amorphous GeTe upon structural relaxation.

#### 2.4.6 Correlations with Peierls(‐like) Distortion

While an enhancement of heavily PD‐like/pyramidal motifs has been found in the current study and in former work,^[^ ^22^ ^]^ other studies found interesting behavior upon aging as well: An increasing bandgap^[^ ^22^ ^]^ and increasing crystallization times^[^ ^69^ ^]^ were found concomitant with the resistance increase upon aging. An intriguing coincidence with the behavior of crystalline GeTe films upon reducing the film thickness becomes apparent: The optical bandgap and the resistivity^[^ ^49^ ^]^ as well as the crystallization temperature^[^ ^49^, ^70^ ^]^ are observed to increase upon decreasing film thickness. Most importantly, those property changes are all accompanied by an increase of the Peierls distortion as a result of the increasing confinement.^[^ ^48^ ^]^ Due to these parallels between scaling effects in crystalline GeTe and aging in its amorphous phase, the observed increase in Peierls(‐like) distortion and the concomitant change to other properties may thus be traced back to the same origin. In GeTe, electron localization is promoted by disorder,^[^ ^15^ ^]^ which is why in amorphous (disordered) GeTe electron localization clearly rules over delocalization. Due to the complete lack of atomic long‐range order in amorphous GeTe, which in the crystal phase promotes the counteracting electron delocalization, localization succeeds in the competition and is the driving force for the structural changes occurring upon aging. Thus, an enhancement of the Peierls‐like distortion, which is a trademark for electron localization, is induced in the amorphous phase upon aging.

Tracing back the origin of resistance drift to this mechanisms, it is quite likely that this process also occurs in other amorphous PCMs. In the crystalline state PCMs (such as GeTe, Sb_2_Te_3_, or pure Sb) are all characterized by a competition between electron localization and delocalization, resulting in a particular property portfolio and classifying these solids as metavalent.^[^ ^45^ ^]^ With the lack of long‐range order in the amorphous phase of this material group the scenario as in amorphous GeTe might be applicable, *i. e*., an increase in Peierls distortion can be hypothesized. While the hypothesis outlined above seems plausible, further experimental evidence is necessary to confirm it.

## 3 Conclusion

Here, we developed an ePDF analysis technique based on electron diffractometry that features a novel background and inelastic scattering correction and is combined with RMC simulations. This way comparable results to X‐ray and neutron scattering are obtained within maximum intervals of up to 19 Å^−1^ in reciprocal space and approximately 10 Å in real space. This TEM‐based electron diffractometry technique of obtaining detailed and reliable PDF data will accelerate structural research in both crystalline and amorphous systems, as TEM is generally more accessible than large scale synchrotron‐ or neutron facility beam time. The ePDF‐analysis software developed can be found here: Extended‐eRDFAnalyser. The resulting data yield even more insight like bond angle distributions, when applying RMC simulations. Combining our modified ePDF analysis with RMC simulations reveals delicate structural rearrangement processes of amorphous GeTe. Namely that upon structural relaxation, the fraction of tetrahedral Ge sites decreases while that of pyramidal/Peierls‐like distorted sites increases, concomitant with an increase of the order parameter *r*_2_/*r*_1_. As usually this increase would be interpreted as an increasing tetrahedral fraction, the current insights resolve this puzzle. Furthermore, the most abundant structural motifs in amorphous GeTe were identified to be heavily Peierls‐like distorted motifs. This structural rearrangement upon vitrification towards more Peierls distortion compared to the crystal is apparently a fingerprint of metavalent solids.

## 4 Experimental Section

### Sample Prepartion

Five amorphous GeTe films of 30 nm thickness had been produced by unheated magnetron sputter deposition at *T* = 293 K. The films were deposited on a commercially available 8 nm thick amorphous carbon film supported by a copper grid (Plano, Germany). Four of the samples had been heated under argon atmosphere to *T* = 378 K (105 °C) for 1.5 h (90 min), 6 h, 24 h, and 96 h, respectively. The last sample was not heated (referred to as as‐deposited). Surface oxidation was minimized by storing the samples in an argon atmosphere between fabrication, annealing, and measurements.

### TEM Diffraction

The films were investigated in an FEI Tecnai TEM by selected area electron diffraction (SAED) with an acceleration voltage of 200 kV and without energy filter. Several SAED patterns at different sample locations had been acquired for each sample and were averaged after the azimuthal integration of the diffraction rings. It was assured that no crystallization occurred (during annealing or due to the electron beam) by inspecting the SAED patterns and the integrated diffraction rings for sharp diffraction spots.

### Software and Analysis

The open‐source software eRDF Analyser ^[^ ^51^ ^]^ had been used for the structure function and PDF calculation, as thoroughly described in Section. SI (Supporting Information). The ability to modify the original MATLAB code was beneficial to enhance the workflow, implement the correction procedure for inelastic electron scattering and add direct analysis tools. The modified code was uploaded and is now available on GitHub (Extended‐eRDFAnalyser), as well as the standalone MATLAB software for calculation of theoretical PDFs from crystallographic information files (CIF2PDF).

The analysis comprises two parts: Trends upon relaxation were extracted based on a qualitative analysis of structural order parameters and, subsequently, these results were corroborated with Reverse‐Monte‐Carlo (RMC) simulations toward a more quantitative understanding. For the RMC simulations, RMCProfile ^[^ ^60^ ^]^ was employed, which had been extended by the developers such that electron atomic form factors could be applied.

## Conflict of Interest

The authors declare no conflict of interest.

## Supporting information

Supporting Information

Supporting Information

## Data Availability

The data that support the findings of this study are available from the corresponding author upon reasonable request.
