# Supplementary material for: Evolution of Short‐Range Order of Amorphous GeTe Upon Structural Relaxation Obtained by TEM Diffractometry and RMC Methods
Source: Adv Sci (Weinh). 2023 Oct 31;10(36):2304323. doi: 10.1002/advs.202304323 (PMC10754132; doi:10.1002/advs.202304323)
Supplement: Supplementary file 1 — Supporting Information [file ADVS-10-2304323-s002.pdf]

ADVANCED SCIENCE
Open Access

Supporting Information

for *Adv. Sci.*, DOI 10.1002/advs.202304323

Evolution of Short-Range Order of Amorphous GeTe Upon Structural Relaxation Obtained by TEM Diffractometry and RMC Methods

Christian Stenz, Julian Pries, T. Wesley Surta, Michael W. Gaultois and Matthias Wuttig**

Supplemental Materials: Evolution of short-range order of amorphous GeTe upon structural relaxation obtained by TEM diffractometry and RMC methods

S-I. INTRODUCTION TO 'ERDF ANALYSER' AND ADDITIONAL IMPLEMENTATIONS

A. General operating principle and definitions

`eRDF Analyser` is an interactive program to compute reduced pair distribution functions (also denoted RDF or $G(r)$). From an input selected area electron diffraction (SAED) pattern the program computes the azimuthal average, reduced structure function $\phi(q)$ and PDF $G(r)$ using an element based analytic approach for the background subtraction. For a more detailed description of the original program than in what follows, please refer to the original paper[S1].

From the 2D diffraction pattern (SAED pattern) and a corresponding calibration factor that translates pixels to reciprocal space length the software computes an azimuthal average around the center of the diffraction rings. By that a 1D diffraction profile $I(q)$ is quantified. Subsequently, an input about the elemental composition is requested. The elemental portions p_i of the composing atomic species i are necessary for extracting the reduced structure function $\phi(q)$:

$$\phi(q) = \left[\frac{I(q) - I_{\text{fit}}(q)}{N \cdot [\sum_i p_i f_i(q)]^2}\right] q, \tag{S1}$$

$$I_{\text{fit}}(q) = N \cdot \sum_i p_i f_i^2(q) + C. \tag{S2}$$

Therein, N is a general scaling factor and C an offset constant. The sum is over the elements in the composition under investigation and the corresponding electron atomic form factors $f_i(q)$ are given by the program according to Kirkland[S2] or Lobato[S3], respectively. The difference between both is insignificant, hence, Kirkland's parameterization has been applied exclusively. By solving a system of equations the only unknown parameters N and C are computed analytically.
Having found $\phi(q)$, the reduced PDF is calculated by numerical integration:

$$G(r) = 4\int_{q_{\text{min}}}^{q_{\text{max}}} [\phi(q) \cdot L(q)] \sin(qr)\, \mathrm{d}q, \tag{S3}$$

whereby $L(q)$ incorporates a damping term to reduce truncation ripples that may be introduced in $G(r)$ due to the finite q_{max}. In the original `eRDF Analyser` an exponential decay function $\exp(-bq^2)$ with damping factor b was used. However, a different damping function was applied in the current work since much better elimination of truncation ripples was found for

$$L(q) = \text{sinc}\left(\pi \frac{q}{q_{\text{max}}}\right) \tag{S4}$$

which describes the damping term introduced by Lorch[S4].
As the structure function $S(q)$ is requisite to obtain the order parameter $S(q_2)/S(q_1)$ its calculation is implemented additionally by the following equation:

$$S(q) = \frac{\phi(q)}{q} + 1. \tag{S5}$$

B. Obtaining the intensity profile

The process of calculating the one-dimensional intensity profile (1D I(pixel)) was enhanced in multiple ways. I(pixel) is here to denote the diffracted intensity as a function of pixels to the center. Later on I(pixel) is translated to $I(q)$ by using the calibration factor ds (*cf.* Sec. S-I D).
Loading of only single diffraction patterns (DPs) is possible in the original program, which is poor from a statistical point of view. To provide statistically more reliable data loading of multiple DPs was implemented. By that, several DPs taken at different locations on the same sample can be averaged and saved to a single diffraction profile I(pixel).
Formerly, the beam stop and the image specifiers in the bottom corners of a DP had to be cut out free-hand in the images each time. As there are many DPs to be processed and to minimize variation in data processing it was advantageous to create a specific mask for the beam stop in a separate image file and to move the mask automatically to the desired position. The mask

therefore was designed to exactly match the shape of the beam stop. Via maximizing the cross-correlation of the DP with the mask the optimum mask position for the mask is found for each DP.
By modifying the input parameters for the DP TIF-files the image layer that contains additional text information is discarded. Hence, the text at the bottom of each DP is no longer needed to be cut manually.
Furthermore, to suppress user interaction completely during the averaging process of several DPs the center has to be found automatically. For the center finding process to start only the first guess had to be provided by a user input. That input was replaced by contrast manipulation of a copy of the DP and using a circular object identifier in MATLAB that provided a very good first guess of the center.

C. Comparing multiple $I(q)$, $\phi(q)$ and $G(r)$ during calculation

To later be able to compare PDFs and deduced quantities of different annealing states directly the import of multiple averaged $I(\text{pixel})$ for simultaneous processing was encoded.

D. Automated calculation of calibration factor ds

For proceeding after $I(\text{pixel})$ is obtained the calibration factor ds is required. ds gives the relation between real space pixels on the detector and reciprocal space distances. It is yielded by recording a DP of a known crystal structure (*e. g.* Au) and identifying the relation between pixel positions of the first few sharp diffraction peaks and the corresponding *hkl* planes. By loading a DP of Au a linear fit of the pixel positions to the known inverse lattice spacing of the crystal is done automatically, *cf.* Fig. S1, and the calibration factor ds is obtained.
The uncertainty on ds provided from the fit routine is negligibly small: An error induced by a false calibration factor which is given by the fit $\sigma_{ds} \sim O(10^{-5})$ (Fig. S1 B) is equivalent to a stretching of the q-axis of the structure function: $q \to aq$ with $a = 1 + \sigma_{ds}$. Firstly, this has no effect on the relative quantity $S(q_2)/S(q_1)$. Secondly, the stretching of the q-axis leads to an alternation in the PDF (Fourier sine transform of $\phi(q)$) as follows:

$$\mathscr{F}[\phi(aq)] = \frac{1}{a} G\left(\frac{r}{a}\right). \tag{S6}$$

Consequently, the error has also no influence on the r_2/r_1-ratio and, ultimately, the relative error on absolute peak position and height is negligibly small:

$$\frac{\Delta r}{r} = \frac{r - \frac{r}{a}}{r} = 1 - \frac{1}{1+\sigma_{ds}} \sim O(10^{-5}), \tag{S7}$$

equivalently for $G(r)$.

E. Correction degree $C_{\mathbf{D}}(r_{\mathbf{co}})$

The correction degree $C_\mathrm{D}(r_\mathrm{co})$ shall give an estimation of the changes experienced by the original structure function due to the applied modification specified by r_co. It is calculated by:

$$C_\mathrm{D}(r_\mathrm{co}) = \frac{\int |\phi_\mathrm{u}(q) - \phi_\mathrm{c}(q, r_\mathrm{co})|\,\mathrm{d}q}{\int |\phi_\mathrm{u}(q)|\,\mathrm{d}q} \tag{S8}$$

$$= \frac{\int |f_\mathrm{cf}(q, r_\mathrm{ro})|\,\mathrm{d}q}{\int |\phi_\mathrm{u}(q)|\,\mathrm{d}q}. \tag{S9}$$

Therefore, $C_\mathrm{D}(r_\mathrm{co})$ is a normalized measure for the absolute difference between uncorrected and corrected structure function (*cf.* Fig. S2).
It is used to find an appropriate range of r_co, such that the correction is large enough to fully remove the distortion from the structure function but simultaneously ensuring not to depress any physical peaks in the resulting PDF. This is best done by inspecting the correction degree as a function of the cutoff distance r_co. Exemplary for all annealing states it is plotted in Fig. S3, where also the resulting evolution of $G(r)$ and $S(q)$ are depicted.

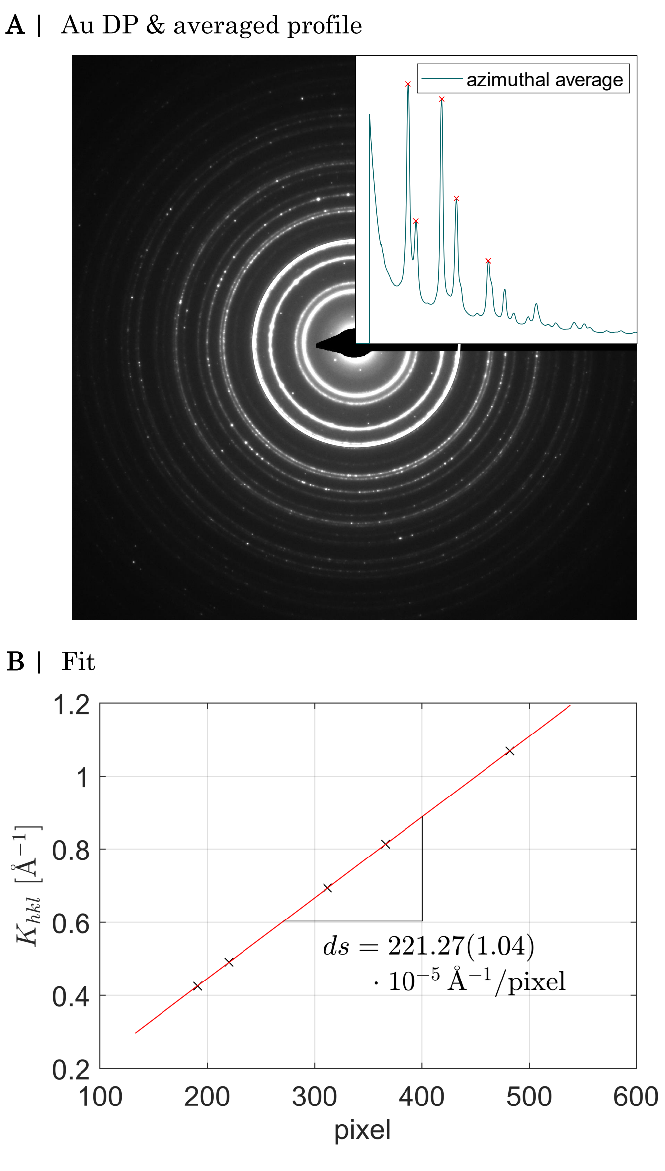

FIG. S1. **Computation of ds. (A)** SAED Pattern (DP) of Au standard sample. The inset shows the averaged intensity I(pixel) with the red crosses marking the peak positions in pixels used for fitting. **(B)** Linear fit of the peak positions (pixels) to the peaks' known reciprocal *hkl* lattice spacing (K_{hkl}) of Au, taken from Tab. S2.

For every investigated sample the correction degree $C_D(r_{co})$ becomes stationary at about $r_{co} = 0.4$ Å. $C_D(r_{co})$ steeply increases for $r_{co} < 0.4$ Å and beyond that the correction degree runs into a saturation. Only after the saturation is reached the main distortion in $S(q)$ is corrected and peak heights shall provide a meaningful measure. The minimally possible separation distance of atoms in most materials is about 1.4 Å or larger, depending on the atomic radii. This value for the correction range r_{co} must not be exceeded. An appropriate cutoff distance is specified to be at an intermediate value of $r_{co} = 1.0$ Å.

F. Uncertainties of Gaussian fits on $S(q)$ and $G(r)$ maxima

To obtain an estimation of the fit uncertainty possibly arising from slightly asymmetric maxima or non-Gaussian behavior each maximum is fitted five times with a varying number of fitting points around the topmost data point. The mean and standard deviation computed from the five fits each give the resulting value (q_i, $S(q_i)$, r_i, $G(r_i)$) and the corresponding fit uncertainty (σ_{fit}), respectively.

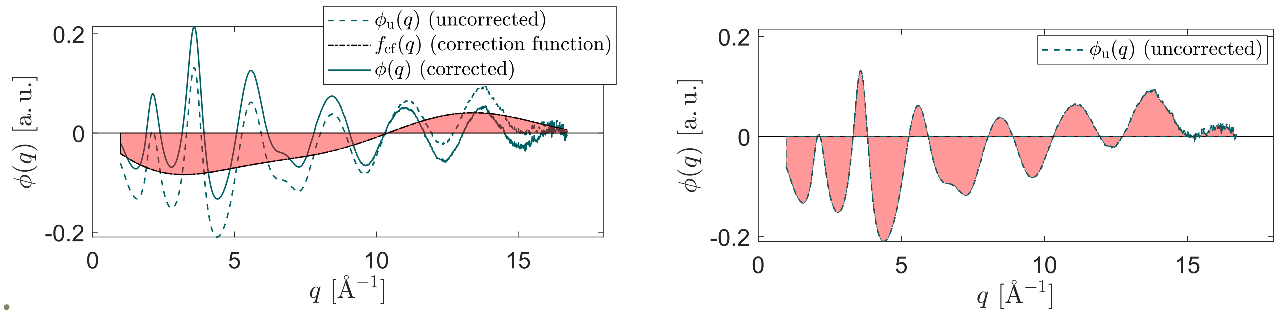

FIG. S2. **Illustration of the quantities contributing to $C_D(r_{co})$.** **LEFT:** The area under the correction function equal to the difference between $\phi_u(q)$ and $\phi_c(q, r_{co})$ is shaded. **RIGHT:** The area under the uncorrected structure function used for normalization is shaded.

G. Evolution of r_i and $S(q_i)$ upon correction

For one sample the evolution of r_i and $S(q_i)$ upon correction shall be analyzed exemplary in the following with special emphasis on the relevant quantities for the order parameters r_1, r_2, $S(q_1)$ and $S(q_2)$ to motivate and justify an averaging over R_{co}.
The low-r_{co} results for the structure function $S(q)$ (Fig. S4, top six graphs) differ from the almost stationary values at higher r_{co}. The results at low-r_{co} values, especially those for $S(q_1)$ and $S(q_2)$ which change by up to 5 %, are not meaningful because the peaks do not oscillate around unity as physically expected. Thus, only after the saturation plateau in $C_D(r_{co})$ is reached a useful meaning is attributed to the quantities. Notice the scale of q_1 and q_2 being much smaller and changes amount to only 0.25 % at most, which is negligibly small.
Inspecting the fit results of the PDF $G(r)$ (Fig. S4, bottom six graphs), the curves as a function of r_{co} seem not to become completely stable at $r_{co} \approx 0.5$ Å contrary to the correction degree $C_D(r_{co})$, but the averaging over R_{co} shall account for these changes. In contrast to the peak heights $G(r_i)$, the alternation in r_2 merely amounts to ~ 0.1 % and in r_1 even only to ~ 0.04 %, which is negligible.

The average final values for a quantity Q are drawn as a straight horizontal line into the graphs of Fig. S4. The dashed lines above and below show the range of uncertainty as a result of the correction procedure $\sigma_{c,Q}$. The colored error bars to each data point in the plots correspond to the uncertainty due to the fit σ_{fit}.

H. Uncertainty on order parameters due to statistical deviations

As by computing the errors in the correction procedure no statistical variation due to measurement inaccuracies is assumed, the estimated total errors have been verified with respect thereto and where modified if required.
To provide statistically more reliable data, averaging of multiple diffraction patterns (DPs) was conducted. Several DPs taken at different locations on each sample were averaged to a single diffraction profile I(pixel). By using the whole procedure extensively explained in the main text the Gaussian fit results of the averaged and the individual I(pixel) as starting points for the calculation were compared. It was found that the results of the averaged DP represents a very good mean value. The statistical uncertainty in the DPs is well covered by the total error established in the preceding section with one exception: The values in the case of the $G(r)$ peak positions r_i statistically vary by a larger extent than the total error σ_{r_i} previously assumed (Eq. 11). In order to account for the statistical variation in DP acquisition the uncertainty for the peak position r_i was altered as follows:

$$\sigma_{r_i} \rightarrow \sqrt{\sigma_{r_i}^2 + (0.05\,\% \cdot r_i)^2}. \quad \text{(S10)}$$

Therefore, the fitting (σ_{fit}) and correction (σ_c) uncertainties become mostly negligible compared to the uncertainty in r_i originating from statistical variations in the DPs.
Ref. S1 and Ref. S5 assume a total uncertainty of 0.3...0.5 % and 0.5 % on r_i, respectively, which is one order of magnitude larger. Due to the thorough considerations regarding uncertainties as a result of the elaborated correction the here established uncertainties exceed the precision of the aforementioned references, where just an upper limit might have been assumed.
According to Gaussian error propagation, the uncertainty on r_2/r_1 and $S(q_2)/S(q_1)$ are calculated from the contributing total errors σ_Q. By that, after reasonable consideration of uncertainties, a well-established error on the measured quantities is found. Based thereon, statements in the data analysis can be made when a change in the order parameters is significant.

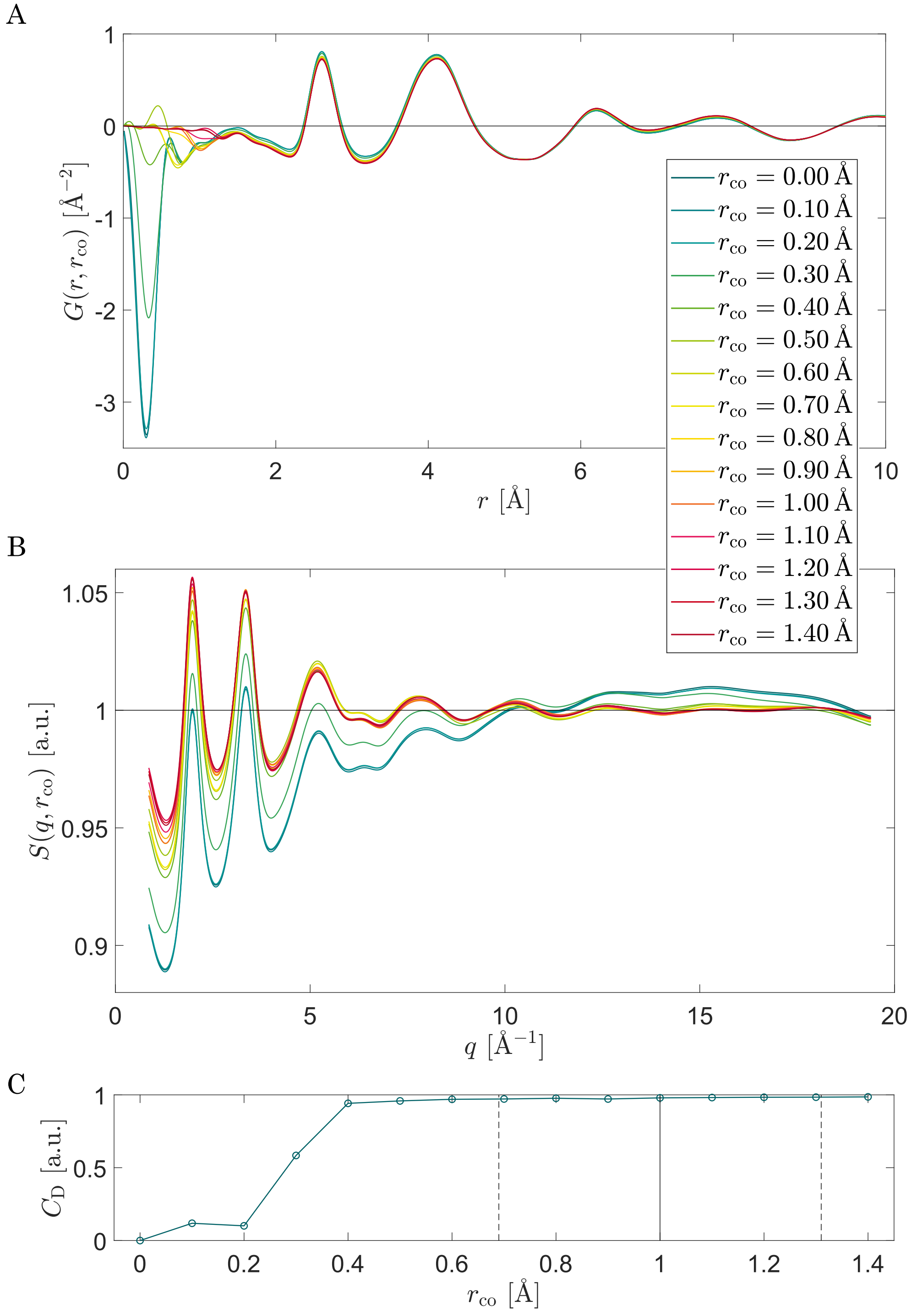

FIG. S3. $G(r)$ and $S(q)$ evolution upon correction. **(A)** Change in $G(r)$ upon increasing r_{co}. The unphysical minimum at low r-values diminishes continuously. **(B)** Change in $S(q)$ upon increasing r_{co}. $S(q)$ only gives reasonable values for the heights of the first two maxima ($S(q_1)$, $S(q_2)$) after an appropriate correction has been conducted and $S(q)$ oscillates well around unity as theoretically expected. **(C)** Correction degree as a function of r_{co}. It saturates after r_{co} reaches a value of 0.4 Å. As explained in the text and indicated by the vertical lines in the plot, quantities are averaged over the marked range of $r_{co} \in [0.7, 0.8, \ldots, 1.3]$.

S-II. COMPARISON OF PDFS OF ZACHARIASEN GLASSES WITH CORRESPONDING CRYSTALLINE ATOMIC DISTANCES

Between amorphous C and crystalline C (graphite) a large agreement of the first two coordination shells becomes apparent (Fig. S5 A). Due to a small shift of the amorphous maxima (a-maxima) to slightly larger distances the amorphous structure seems slightly less dense. The measured curve is considered to show correct principal peak positions.
In Fig. S5 B amorphous Si is under investigation. It shall be noticed that the small pre-peak at ~ 1.5 Å is not attributed to cutoff ripples or similar artifacts but rather to Si-O bonds that possibly occurred to a small extent due to surface oxidation (the sample was not kept under Argon atmosphere between fabrication and measurement in contrast to the GeTe samples). Nonetheless, a large accordance is observed between the SRO of the amorphous and crystalline phases. Even the third crystalline peak (c-peak) may be slightly represented in the amorphous case by the asymmetric minimum. Therefore, also for a-Si a proper PDF that matches the expected peak positions was established after applying the correction procedure.
In Fig. S6 A & B, the α- and β-structure of crystalline Si_3N_4 are compared to the amorphous PDF. The c-peaks and a-peaks in the first coordination shell match almost perfectly in both cases. Although in the second nearest neighbor distance a large variation occurs in the crystalline structures, again, a generally good agreement between the first two coordination shells is observed.
Therefore, all investigated glasses show a large agreement of the first two coordination shells with their corresponding crystal structure, as it is expected for Zachariasen glasses. Additionally to the good accordance of the measured PDF of c-$Ge_2Sb_2Te_5$ with the theoretical atomic distances (*cf.* Fig. 2 B & C in the main text), the agreement of experimentally measured distances for the nearest- and next-nearest neighbors with the expected distances for the crystals indicates the validity of the data treatment applied in this work to obtain amorphous PDFs that reproduce peak positions correctly.

S-III. COMPARISON OF ACQUIRED $S(q)$ AND $G(r)$ DATA TO OTHER GETE STUDIES

It is checked that $S(q)$ and $G(r)$ of amorphous GeTe are consistent with experimental data, as well as with molecular dynamics (MD) simulation data, *cf.* Fig. S7. Our data (like any other experimental PDF study of amorphous GeTe) differs from simulated results to some extent, specifically the first nearest-neighbor (NN) distance is distinct. For melt-quenched MD simulations NN distances are reported to be around 2.8 Å and for experimental as-deposited amorphous GeTe it is around 2.6 Å (which was already addressed in the main text introduction).

S-IV. EXPERIMENTAL DATA

Fig. S8 A & B show the corrected structure functions $S(q)$ for all annealing states of GeTe and their differences $\Delta S(q)$ with respect to the as-dep. sample, respectively. In Fig. S9 A & B the acquired real-space data $G(r)$ and the changes $\Delta G(r)$ with respect to the as-dep. state are presented, respectively.

S-V. RMC SIMULATION DETAILS

A. SiO_2

For the SiO_2 simulations a starting configuration was supplied by Tucker *et al.* comprised of 6144 atoms in total. Its number density is 0.0667 $Å^{-3}$. Minimum bond distances for Si-Si (2.7 Å), Si-O (1.4 Å) and O-O (2.3 Å) have been applied. Furthermore, a polyhedral restraint has been set to keep the tetrahedral SiO_4 units during the simulations.
The resulting fits for reciprocal- and real-space show a good agreement to the experimental data, *cf.* Fig. S10 A & B. Also the partial PDFs (Fig. S10 C) are in line with former SiO_2 RMC simulations performed on data from neutron or X-ray beam lines.
It was discovered that particularly the Si-Si-Si bond angle distribution (BAD) can be influenced by using different starting configurations for the RMC simulation. Simulations starting from a crystalline structure like the α-SiO_2 or Coesite-SiO_2 structure converged to similarly good agreement as those simulations from the amorphous starting configuration. The only exception was the Si-Si-Si BAD in that case showing a broadened and bimodal curve which was closer to the Keen 1997 result. The highest peak in the Si-Si-Si BAD was in that case centered at $\theta = 90°$, which is not surprising, since the crystalline structures also possesses many rectangular Si-Si-Si configurations.

B. GeTe

The GeTe RMC simulations were conducted multiple times for different starting configurations and different fitting details until the simulation converged with the experimental data. Fits were conducted with both the corrected structure function (Eq. 5) and the PDF (Eq. 6) simultaneously. Best fit results were found for minimum atomic distances of 2.4 Å for both Ge-Ge and Ge-Te bonds as also applied by Stellhorn *et al.*[S9] and 3.0 Å for Te-Te (which is slightly larger as compared to 2.8 Å applied by Stellhorn *et al.* because otherwise an undesired accumulation of Te-Te bond distances emerged at the cutoff distance of 2.8 Å). Atom swaps between Ge and Te sites are allowed and occur with 10 % chance throughout the simulation instead of an atom displacement. Starting with a tetrahedral or a heavily Peierls distorted crystal configuration, respectively, with atomic number densities of 0.033270 $Å^{-3}$ and a size of 8000 atoms had no significant effect on fit quality or extracted quantities. For the sake of better statistics, four simulation runs (one with the Peierls distorted and the other three with the tetrahedral starting configuration) were conducted for each of the five annealing states. Each run was analyzed individually with respect to their bond-angle distribution, distribution of nearest-neighbors and amount of tetrahedral Ge centers. Therefore, Fig. 7, 8 and 10 in the main text show the weighted mean and uncertainty of the four runs for each annealing state.

S-VI. STRUCTURAL MOTIF IDENTIFICATION USING DISTANCE DISTRIBUTIONS OF NEAREST NEIGHBORS

To identify the relative proportion of several structural motifs in amorphous GeTe, the distance distribution of nearest-neighbors (DDNN) is examined. That is, for every atom in the system, the nine nearest neighbors are identified and their respective distance to the central atom. Based on the neighbor identity (*e. g.* ID = 4 corresponds to the fourth nearest neighbor), the probability distribution for the distance to the center can be calculated with regard to the ID.

Since the three structural motifs (tetrahedral, octahedral, and heavily Peierls distorted) are to be distinguished, the signatures of these structures were identified first. To this end, the DDNN has been computed for a tetrahedral, an octahedral and a heavily Peierls distorted crystal lattice. All the atoms were randomly shifted by a small amount to ensure a narrow Gaussian distribution around the stable atom positions as expected for any disordered system. In Fig. S14 the fingerprint of each motif becomes apparent: In a four-fold tetrahedral structure (Fig. S14 A) the four nearest neighbors of each atom (*i. e.* NN ID = $1 \ldots 4$) align at a short distance $d/d_c < 1$ ($d_c = 3.00605$ Å is the crystalline GeTe bond length), while the higher order NNs with ID > 4 possess an increased bond length. For the Peierls distorted structure (Fig. S14 B) the change from short bond to long bond already occurs from ID = 3 to ID = 4. Contrary, the cubic structure (Fig. S14 C) shows six short bonded NNs before the second coordination shell with its long bonds is occupied. Thus, with the 4th and 5th ID distributions the three structures can be discerned distinctively. Since the amorphous GeTe structure possesses a diverse multitude of structural motifs the DDNN will be a superposition of all three signatures. Fig. S15 shows the DDNNs for the Ge centers where the focus is on. Fitting double Gaussian curves of the form

$$\sum_i \frac{A_i}{\sqrt{2\pi} w_i} \exp\left(-\frac{(d-\mu_i)^2}{2w_i^2}\right) \tag{S11}$$

to the 4th and 5th NN-distribution around Ge atoms provides a measure for the fraction of PD-like, tetrahedral and cubic/defective octahedral motifs of Ge centers. The scaling factors A_i are directly proportional to the area under the Gaussian. Thus, $A_1 = A_{\mathrm{PD}}$ (*cf.* Fig. S15) represents a measure for the number of long bonds on the 4th NN and, hence, a relative measure for PD-like or pyramidal motifs with CN = 3. $A_2 = A_{\mathrm{cub}}$ is a measure for the short bonds on ID 5 and shall represent those motifs which are five- or six-fold coordinated, *i. e.* cubic motifs. A_3 reflects the amount of four-, five- and six-fold coordinated Ge atoms, so by subtracting A_2 from A_3 the amount of 4-fold motifs is left. Therefore, $A_3 - A_2 = A_{\mathrm{tetra}}$ measures the amount of tetrahedral Ge units. The probability P_i of each of the three motifs is then calculated by normalization:

$$P_x = \frac{A_x}{\sum_y A_y} \qquad x, y = \text{PD, cub, tetra.} \tag{S12}$$

A clear advantage of this method is the redundancy of defining a precise cutoff distance which might not be appropriate for a system like a-GeTe possessing a large diversity of intermediate bond lengths.

S-VII. CIF-SOURCES

The sources and references for the Crystallographic Information Files (CIFs) used in this work can be found in Tab. S2.

References

[S1]J. Shanmugam, K. B. Borisenko, Y.-J. Chou, and A. I. Kirkland, "eRDF Analyser: An interactive GUI for electron reduced density function analysis," SoftwareX **6**, 185–192 (2017).

[S2]E. J. Kirkland, *Advanced Computing in Electron Microscopy* (2010).

[S3]I. Lobato and D. Van Dyck, "An accurate parameterization for scattering factors, electron densities and electrostatic potentials for neutral atoms that obey all physical constraints," Acta Crystallographica Section A: Foundations and Advances **70**, 636–649 (2014).

[S4]E. Lorch, "Neutron diffraction by germania, silica and radiation-damaged silica glasses," Journal of Physics C: Solid State Physics **2**, 229 (1969).

[S5]S. Wei, M. Stolpe, O. Gross, W. Hembree, S. Hechler, J. Bednarcik, R. Busch, and P. Lucas, "Structural evolution on medium-range-order during the fragile-strong transition in $Ge_{15}Te_{85}$," Acta Materialia **129**, 259–267 (2017).

[S6]S. Kohara, K. Kato, S. Kimura, H. Tanaka, T. Usuki, K. Suzuya, H. Tanaka, Y. Moritomo, T. Matsunaga, N. Yamada, Y. Tanaka, H. Suematsu, and M. Takata, "Structural basis for the fast phase change of $Ge_2Sb_2Te_5$: Ring statistics analogy between the crystal and amorphous states," Applied Physics Letters **89**, (201910)1–3 (2006).

[S7]J. R. Stellhorn, S. Hosokawa, B. Kaiser, K. Kimura, N. Boudet, N. Blanc, H. Tajiri, S. Kohara, and W.-C. Pilgrim, "The Structure of the Amorphous (GeTe)1−x(Sb2Te3)x System and Implications for its Phase-Change Properties," Zeitschrift für Physikalische Chemie **235**, 141–167 (2021).

[S8]G. E. Ghezzi, J. Y. Raty, S. Maitrejean, A. Roule, E. Elkaim, and F. Hippert, "Effect of carbon doping on the structure of amorphous GeTe phase change material," Applied Physics Letters **99**, 151906 (2011).

[S9]J. R. Stellhorn, S. Hosokawa, W. Pilgrim, N. Blanc, N. Boudet, H. Tajiri, and S. Kohara, "Short- and intermediate-range order in amorphous GeTe," Physica Status Solidi (B) **253**, 1038–1045 (2016).

[S10]Y. Chen, L. Sun, Y. Zhou, G. M. Zewdie, V. L. Deringer, R. Mazzarello, and W. Zhang, "Chemical understanding of resistance drift suppression in Ge–Sn–Te phase-change memory materials," Journal of Materials Chemistry C **8**, 71–77 (2019).

[S11]V. L. Deringer, W. Zhang, M. Lumeij, S. Maintz, M. Wuttig, R. Mazzarello, and R. Dronskowski, "Bonding Nature of Local Structural Motifs in Amorphous GeTe," Angewandte Chemie International Edition **53**, 10817–10820 (2014).

[S12]J. Y. Raty and M. Wuttig, "The interplay between Peierls distortions and metavalent bonding in IV-VI compounds: comparing GeTe with related monochalcogenides," Journal of Physics D: Applied Physics **53**, 234002 (2020).

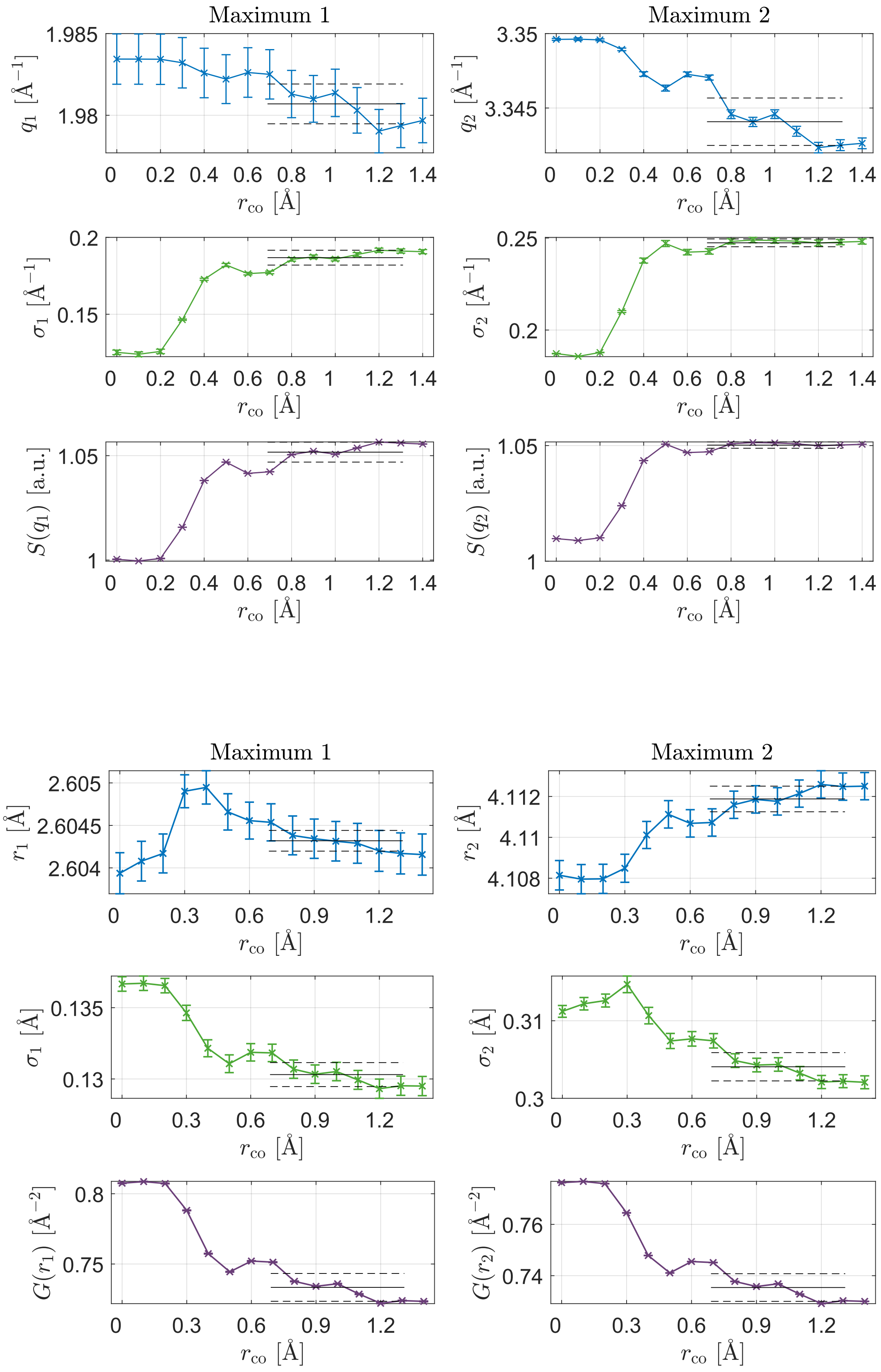

FIG. S4. Evolution of the fit results of the first two $S(q)$ (top) and $G(r)$ (bottom) peaks upon correction.

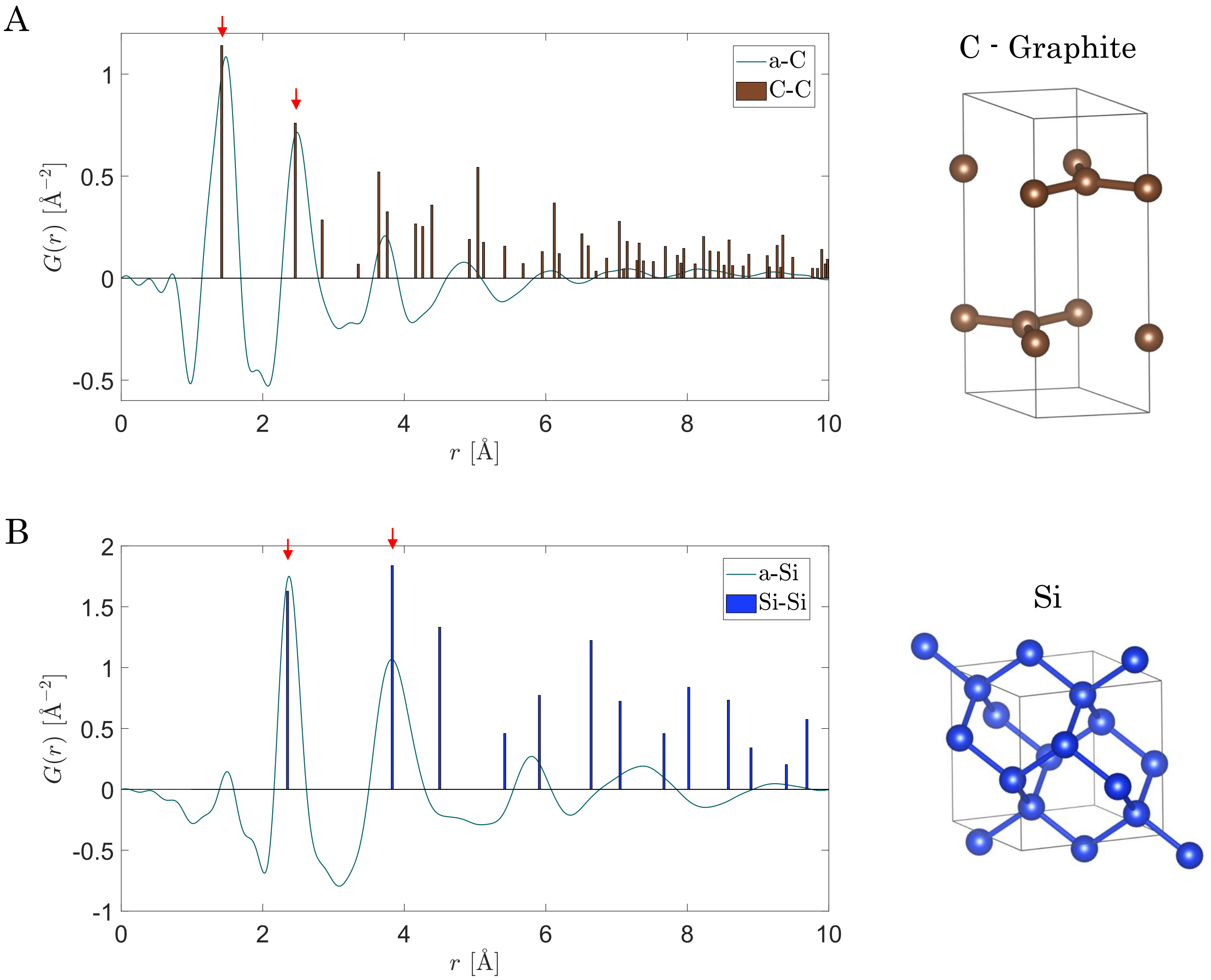

FIG. S5. PDFs of the Zachariasen glasses C and Si compared to the corresponding crystalline models. The crystalline distances have been calculated from the CIFs which are listed in Tab. S2.

TABLE S1. Partial and total coordination numbers for the different annealing states. Cutoff distances are 3.0 Å (Ge-Ge), 3.2 Å (Ge-Te) and 3.4 Å (Te-Te), which are chosen close to the crossover from the bonding to antibonding character of the respective bond (3.05 Å, 3.16 Å, 3.5 Å)[S10]. The statistical uncertainty from the four individual simulations per annealing step amounts to 0.005 for Ge-Te, Te-Ge and Te-Te CNs. No significant changes can be observed upon annealing. The statistical uncertainties for the Ge-Ge CN are shown in Fig. S11. A minor decreasing trend becomes evident from the fit.

	**as-dep.**	**1.5 h**	**6 h**	**24 h**	**96 h**
CN(Ge-Ge)	1.453	1.435	1.440	1.438	1.435
CN(Ge-Te)	2.488	2.520	2.503	2.513	2.478
CN(Te-Ge)	2.488	2.520	2.503	2.513	2.478
CN(Te-Te)	0.230	0.213	0.223	0.213	0.223
N_{tot}(Ge)	3.941	3.955	3.943	3.951	3.913

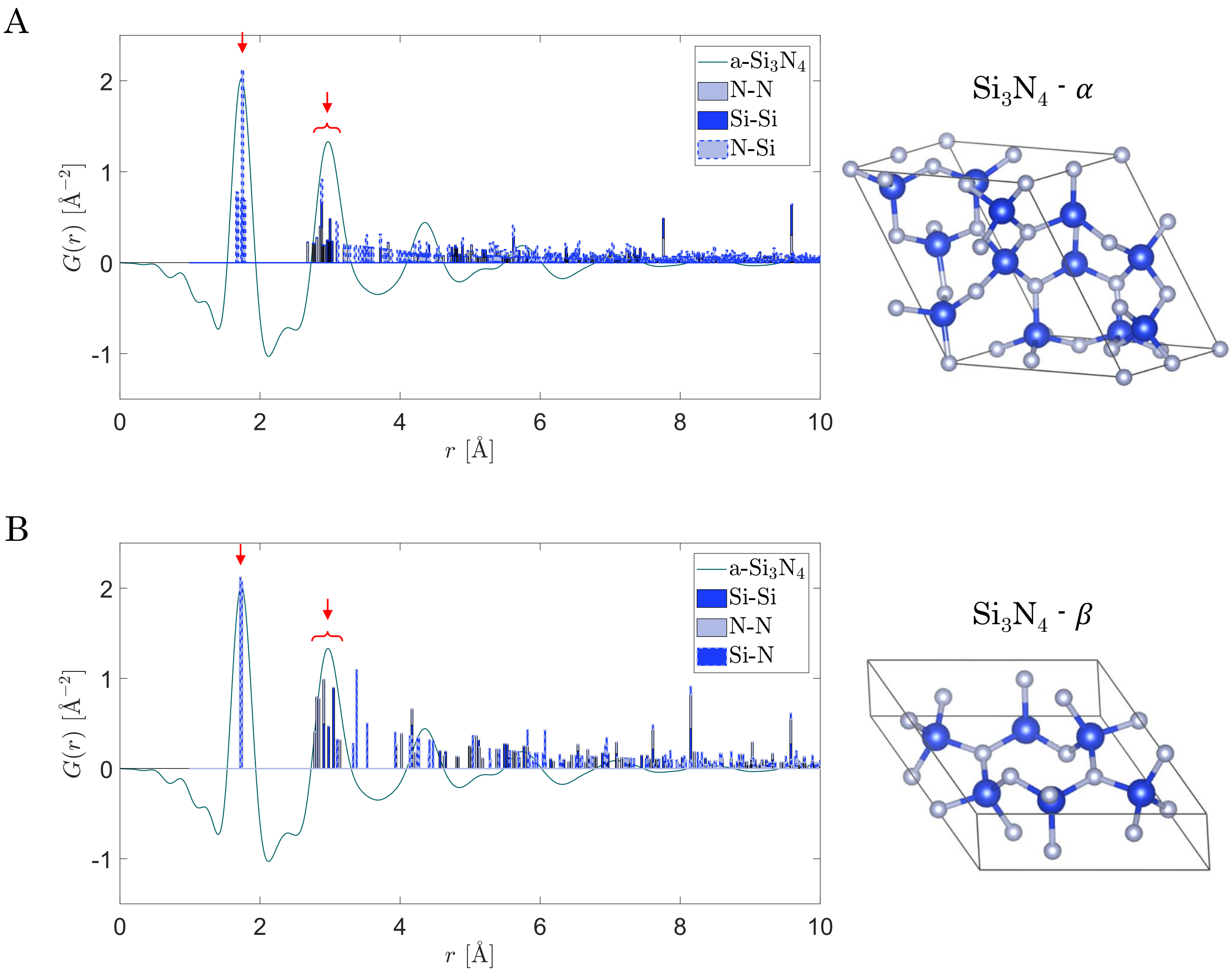

FIG. S6. PDFs of the Zachariasen glass Si_3N_4 compared to the corresponding crystalline models. The crystalline distances have been calculated from the CIFs which are listed in Tab. S2.

TABLE S2. **Sources for Crystallographic Information Files (CIFs).** HT and PD correspond to high temperature and the Peierls distortion ratio (r_{long}/r_{short}), respectively. The Springer Dataset IDs redirect to "http://materials.springer.com/isp/crystallographic/docs/sd_#" where # indicates the corresponding ID. To find sources from ICSD follow https://icsd.fiz-karlsruhe.de/search/basic.xhtml.

**Composition**	**Springer Dataset ID**	**ICSD Collection Code**	**Else**
Au	1628823		
C (Graphite)		230104	
Ge	0549824		
GeTe HT (cubic)			S12
GeTe PD=1.36			S12
$Ge_2Sb_2Te_5$ (cubic)		159908	
$Ge_2Sb_2Te_5$ (rhombohedral)		188968	
Si	0304951		
Si_3N_4 (α)	0532259		
Si_3N_4 (β)	0532265		
SiO_2 (α)	1520590		

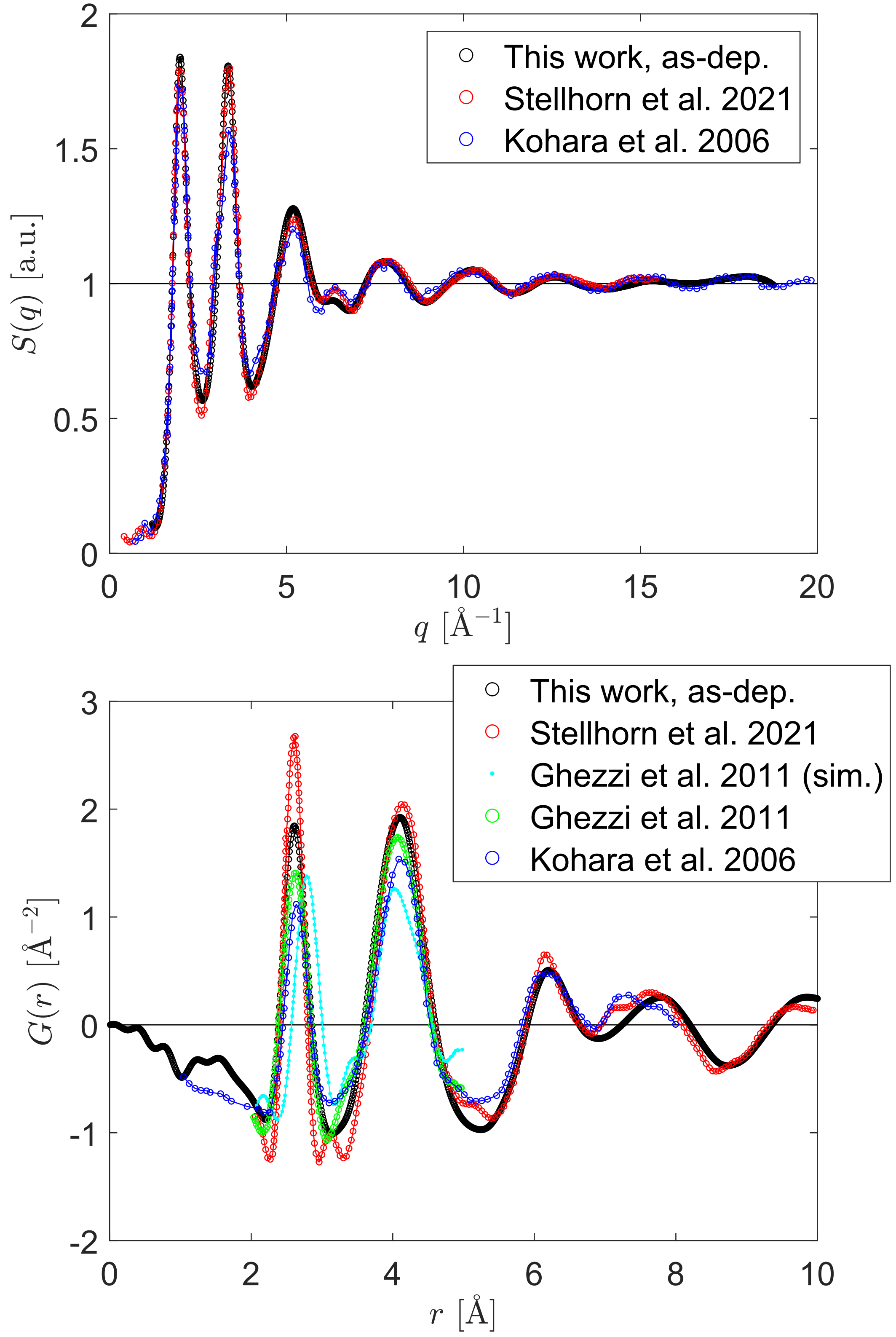

FIG. S7. **Comparison of S(q) and G(r) data for amorphous GeTe taken in this study and in literature.** $S(q)$ is compared to experimental data (X-ray studies) from Refs. S6 & S7. $G(r)$ is additionally compared to data from Ref. S8, which also includes data from a molecular dynamics simulation (cyan curve). Our data (black) are in good agreement with the reported experimental data which only shows slight deviations that are within the expected range for different experimental setups. It is noticeable that $G(r)$ computed from molecular dynamics simulations shows a significantly larger first nearest-neighbor distance compared to the experimental data.

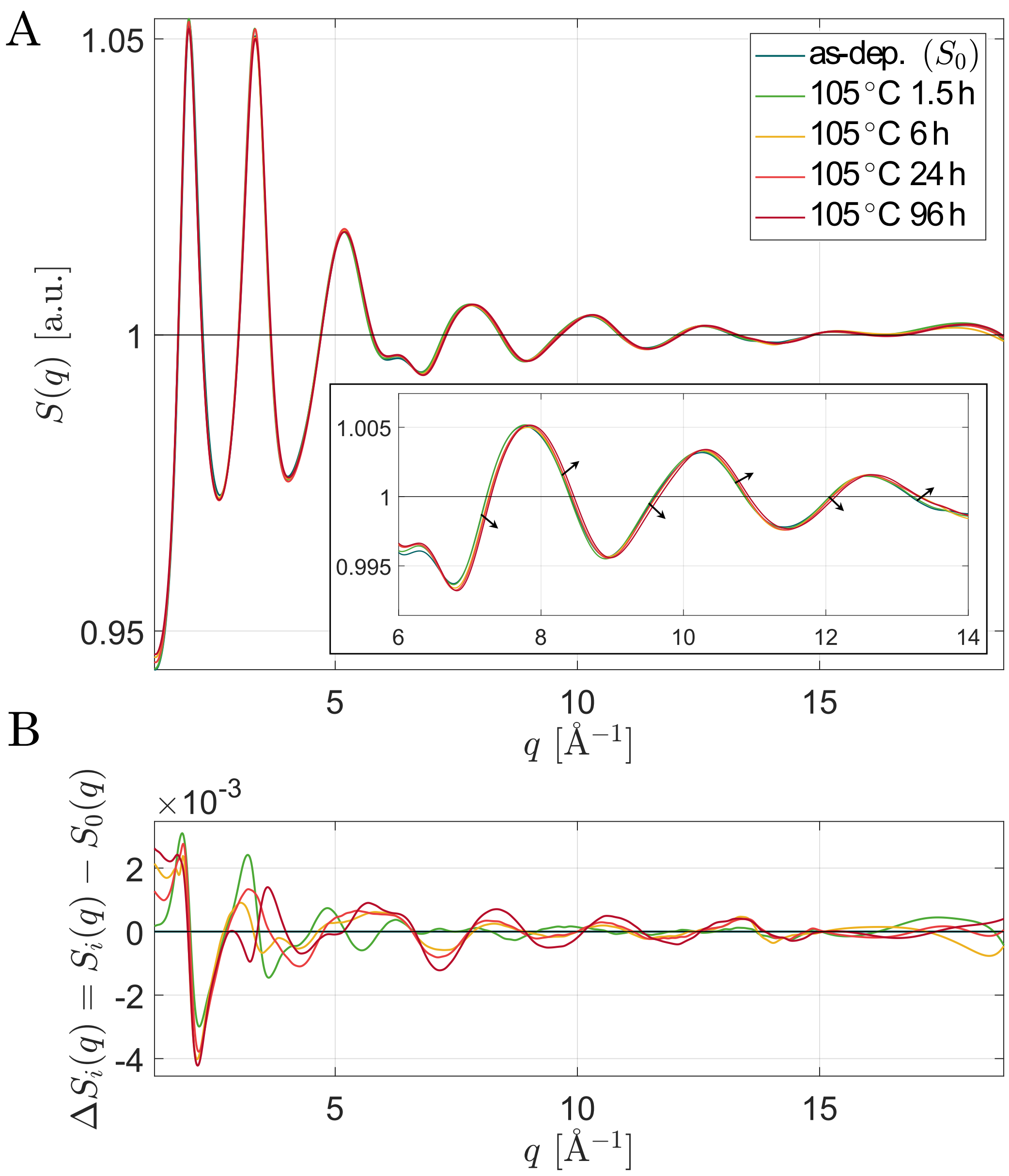

FIG. S8. **(A)** Structure function $S(q)$ for all annealing states after the correction procedure. In the enlargement (inset) a clear continuous shift of the extrema towards larger q-values can be observed upon relaxation. **(B)** Difference of the structure functions with respect to the as-dep. state.

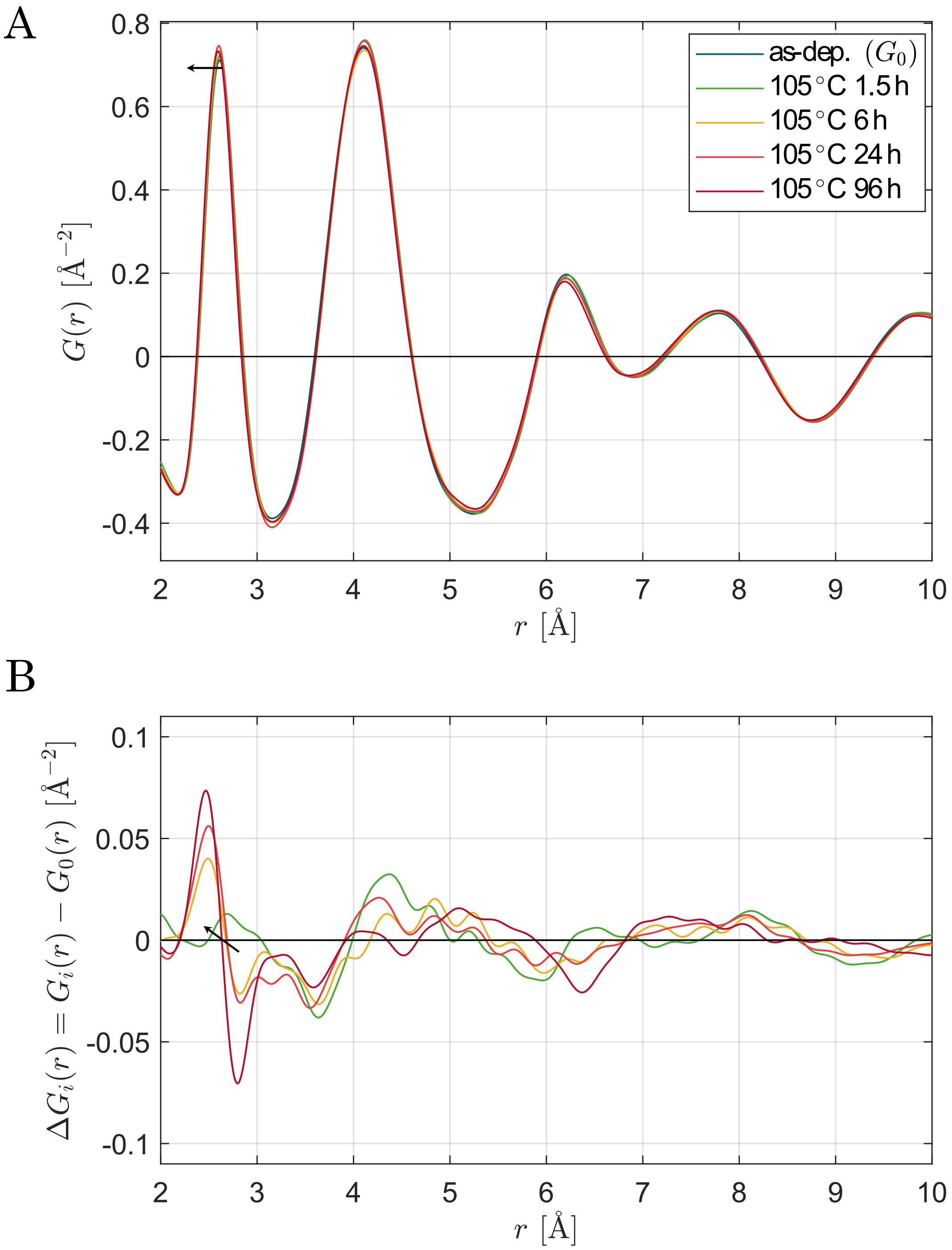

FIG. S9. **(A)** $G(r)$ for all annealing states. **(B)** Difference of $G(r)$ with respect to the as-dep. state.

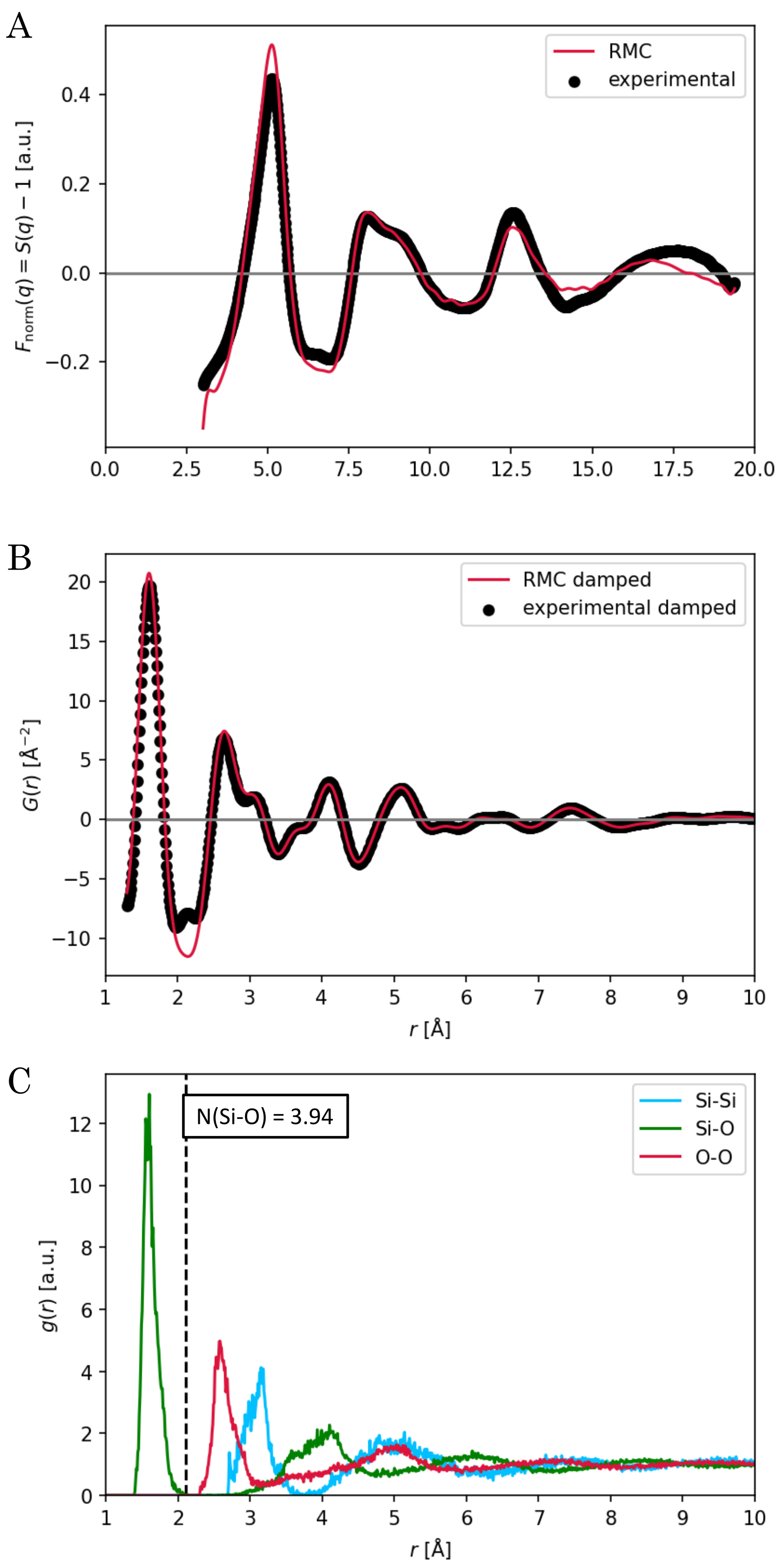

FIG. S10. **RMC fits of SiO_2.** **(A)** Structure function $F_{\mathrm{norm}}(q) = S(q) - 1$. **(B)** Pair distribution function $G(r)$ computed from the structure functions in A with Lorch damping applied. **(C)** Partial pair correlation functions. The coordination number for oxygen around Si centers is shown for a cutoff distance of 2.1 Å.

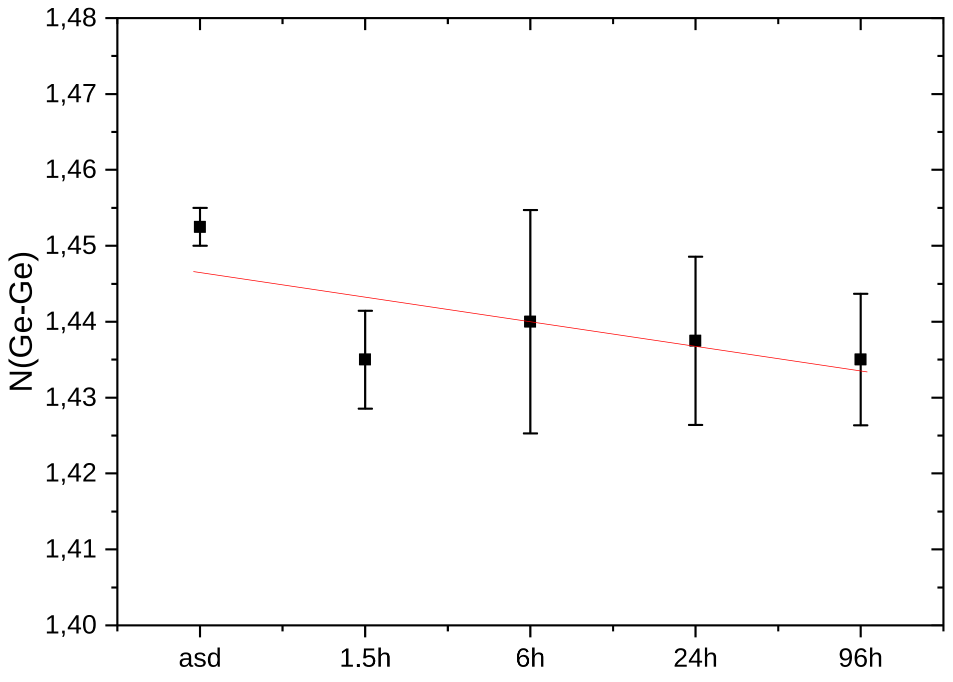

FIG. S11. **Partial Ge-Ge coordination numbers upon annealing.** A slight decreasing trend upon annealing can be inferred.

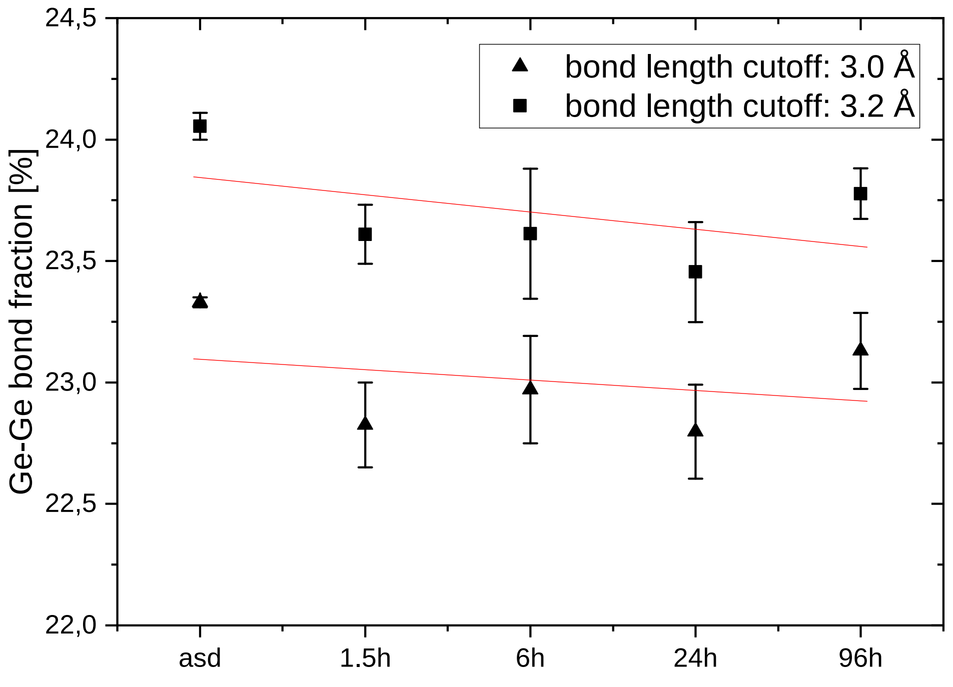

FIG. S12. **Homopolar Ge-Ge bond fraction for two different bond length cutoff distances.** The fraction of Ge-Ge bonds is calculated with respect to all bonds (defined via the bond length cutoff) where at least one Ge atom is involved. There are around 23 % and 24 % of homopolar Ge bonds for a bond length cutoff distance of 3.0Å and 3.2Å, respectively. A minor decreasing trend upon annealing can be observed from the linear fit, which is in congruence with CN(Ge-Ge) (*cf.* Fig. S11).

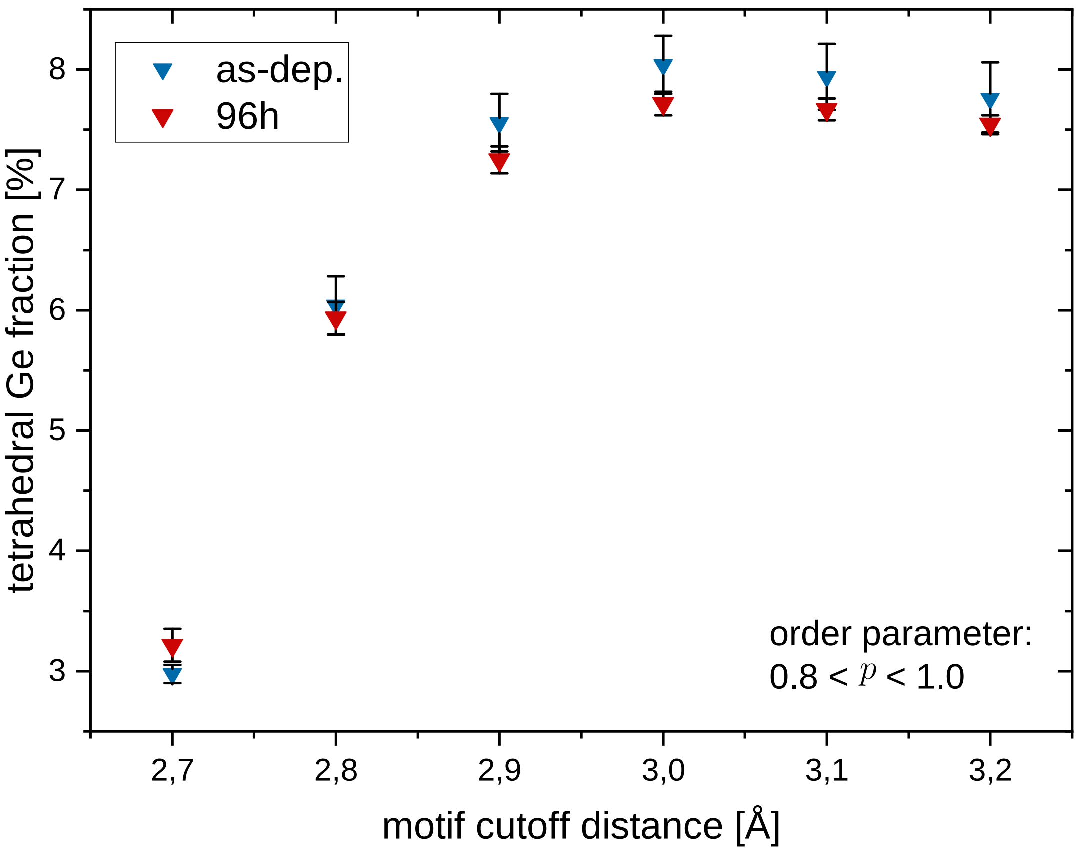

FIG. S13. **Fraction of tetrahedral Ge centers in the as-deposited and 96 h state computed from the order parameter p as function of the applied cutoff distance.** From the pronounced increase of the tetrahedral fraction from a cutoff distance of 2.7 Å towards 3.0 Å it becomes obvious that most tetrahedral Ge centers are bonded to their adjacent atoms within a bond length between 2.7 Å and 3.0 Å. The slight decreasing trend for a cutoff larger than 3.0 Å is because a part of the motifs that were counted as 4-fold tetrahedral might be counted as 5-fold for larger cutoff distances, instead.

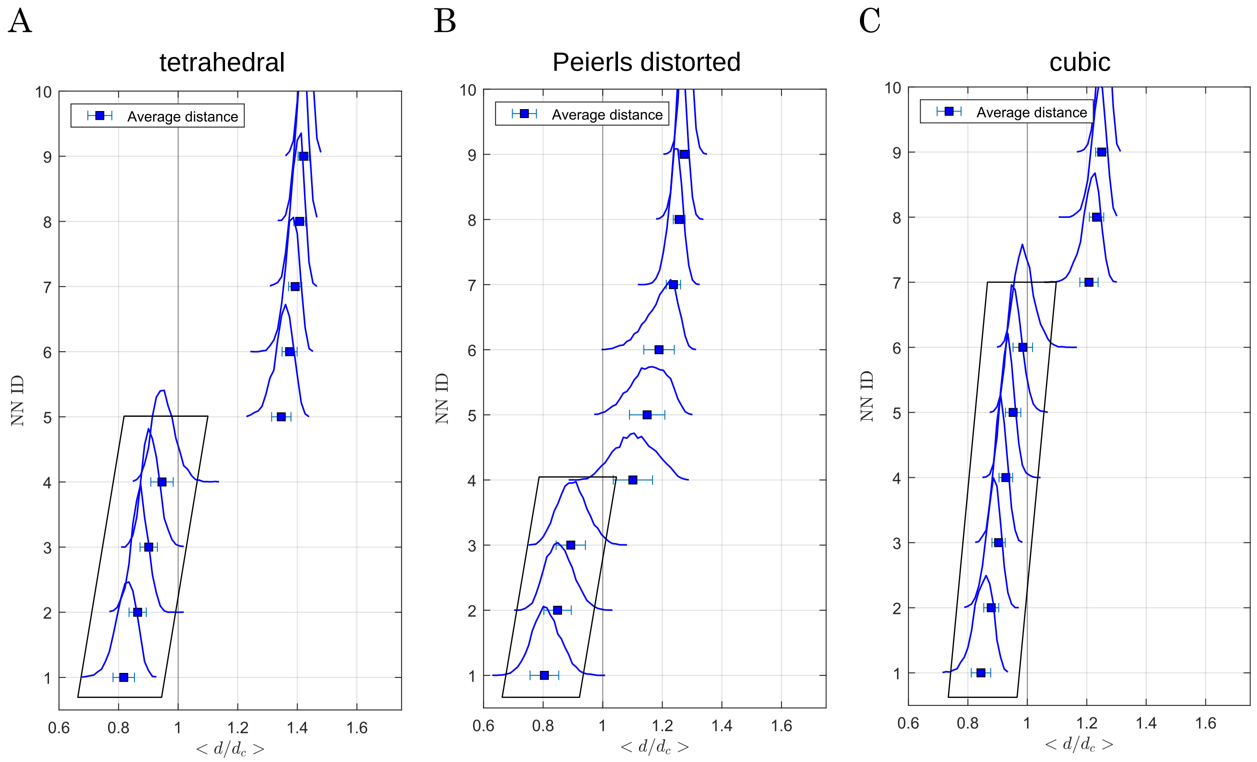

FIG. S14. **Distance distribution of nearest neighbors (DDNN) for the tetrahedral (A), heavily Peierls distorted (*PD*-ratio $= 1.36$) (B), and the perfectly cubic (C) structure.** The distances d are normalized to the crystalline bond length of GeTe with $d_c = 3.00605$ Å. The underlying tetrahedral structure was scaled to an average bond length of 2.66 Å. That bond length is based on bond-weighted distribution functions for tetrahedral motifs in a-GeTe[S11]. Since the bond lengths are slightly different for homo- and heteropolar bonds the here determined fraction of homopolar Ge-bonds (≈23.5 %) was also taken into account. Analogously, the cubic structure was scaled to an average bond length of 2.76 Å. For the PD-structure the same scaling factor has been used as for the perfectly cubic structure.

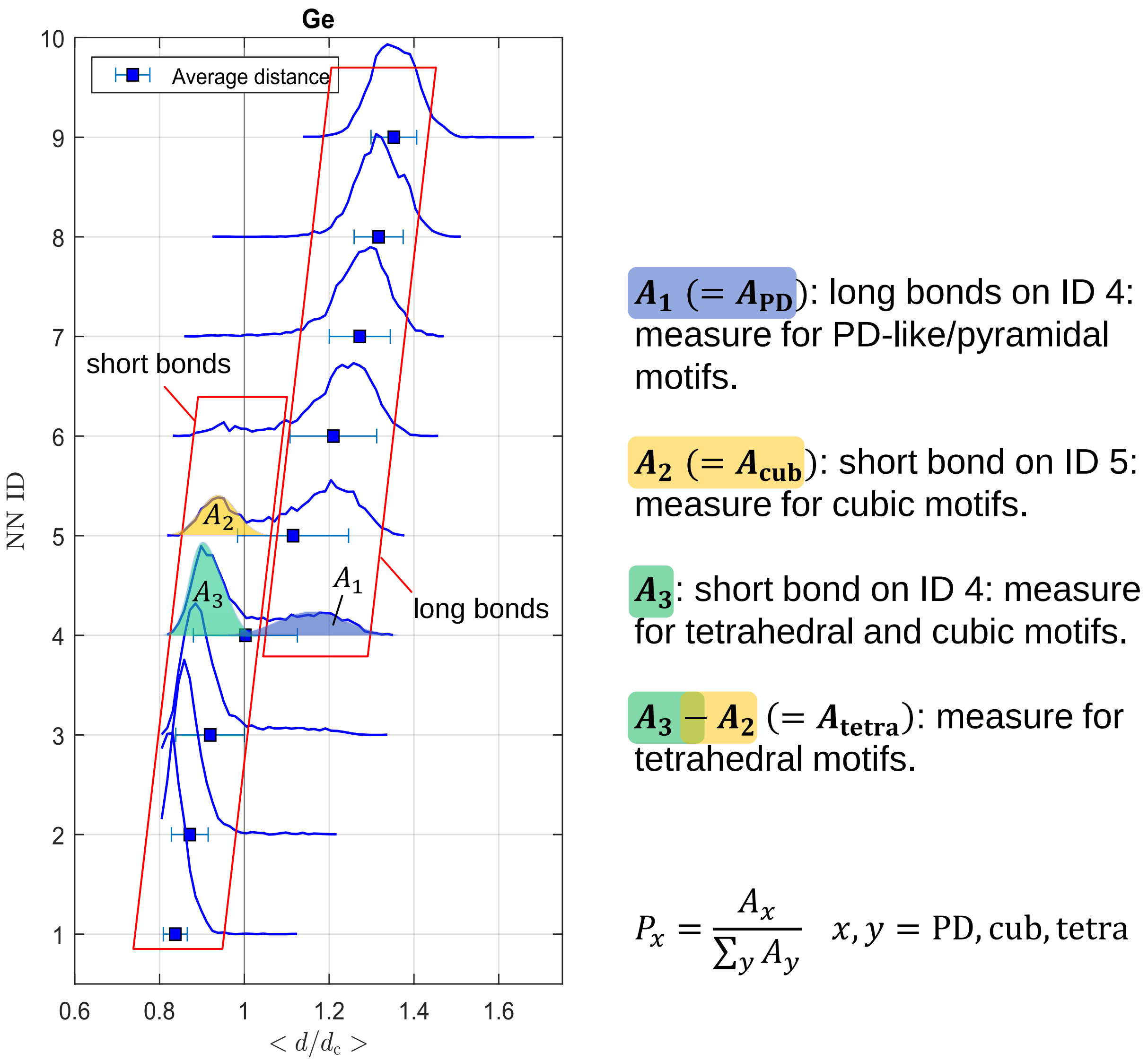

FIG. S15. **Exemplary distance distribution of nearest neighbors (DDNN) for Ge-centers in amorphous GeTe and how a measure for the fraction of Ge-centered motifs can be inferred.** The distances d are normalized to the crystalline bond length of GeTe with $d_c = 3.00605$ Å.

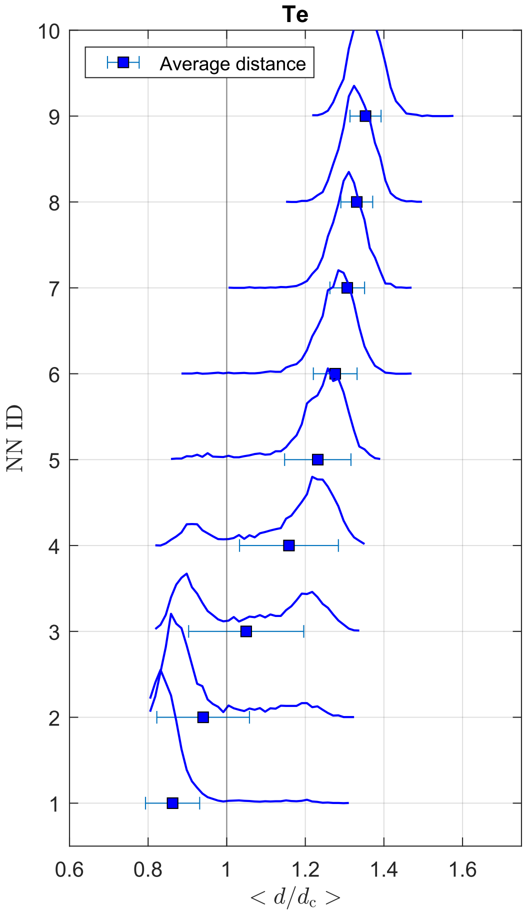

FIG. S16. **Exemplary distance distribution of nearest neighbors (DDNN) for Te-centers in amorphous GeTe.** The distances d are normalized to the crystalline bond length of GeTe with $d_c = 3.00605$ Å. From the Te centered DDNN it becomes apparent that there are similar probabilities for the third NN to appear at a short and at a long bond distance, while the fourth NN is much more likely to be long bonded. *I. e.* many Te atoms are 2-fold (Te^{II}) or 3-fold (Te^{III}) coordinated.
